# Robust real-time 3D imaging of moving scenes through atmospheric obscurant using single-photon LiDAR

**DOI:** 10.1038/s41598-021-90587-8

**Published:** 2021-05-27

**Authors:** Rachael Tobin, Abderrahim Halimi, Aongus McCarthy, Philip J. Soan, Gerald S. Buller

**Affiliations:** 1 School of Engineering and Physical Sciences, Heriot-Watt University, Edinburgh, EH14 4AS UK; 2 Defence Science and Technology Laboratory, Porton Down, Salisbury, SP4 0LQ UK

**Keywords:** Single photons and quantum effects, Imaging techniques, Imaging and sensing

## Abstract

Recently, time-of-flight LiDAR using the single-photon detection approach has emerged as a potential solution for three-dimensional imaging in challenging measurement scenarios, such as over distances of many kilometres. The high sensitivity and picosecond timing resolution afforded by single-photon detection offers high-resolution depth profiling of remote, complex scenes while maintaining low power optical illumination. These properties are ideal for imaging in highly scattering environments such as through atmospheric obscurants, for example fog and smoke. In this paper we present the reconstruction of depth profiles of moving objects through high levels of obscurant equivalent to five attenuation lengths between transceiver and target at stand-off distances up to 150 m. We used a robust statistically based processing algorithm designed for the real time reconstruction of single-photon data obtained in the presence of atmospheric obscurant, including providing uncertainty estimates in the depth reconstruction. This demonstration of real-time 3D reconstruction of moving scenes points a way forward for high-resolution imaging from mobile platforms in degraded visual environments.

## Introduction

The Light Detection and Ranging (LiDAR) technique often uses the time-of-flight (ToF) information of a reflected optical signal to determine the distance to an object^1^. Compared to Radio Detection and Ranging (RADAR) approaches, LiDAR systems are generally capable of higher resolution imaging of objects at long ranges due to the use of much shorter wavelengths^2^. The modular and compact nature of current LiDAR system designs has enabled them to be deployed in a variety of ranging and imaging applications, such as airborne platforms^3–5^ and vehicle navigation systems^5–7^.

More recently, the time-correlated single-photon counting (TCSPC) technique has been employed in prototype three-dimensional imaging LiDAR systems for sensing in demanding scenarios, due to its high sensitivity^8,9^. While some conventional ToF LiDAR systems based on linear avalanche photodiode (APD) detector technologies are capable of providing high-resolution images at long ranges in clear conditions, they lack however the shot-noise limited sensitivity provided by the time-correlated single photon detection approach. The high sensitivity exhibited by single-photon detectors, such as superconducting nanowire detectors (SNSPDs)^10–13^ or single-photon avalanche diode (SPAD) detectors, allow the use of low average optical output power levels for scene illumination. Typically, both detector types are capable of picosecond timing, permitting excellent surface-to-surface resolution that is not readily achievable using an analogue optical detector approach^12,14,15^. SPAD detectors are the most commonly used single-photon detectors for remote sensing applications as they are capable of operation near room temperature, typically in Peltier-cooled packages. Single-pixel SPAD detectors have been used to image a scene by scanning in a point-by-point manner in the visible, near-infrared and short-wave infrared regions^16–20^. SPAD detector arrays have also been used in large pixel formats (e.g., 32 × 32, 128 × 64) that can offer high spatial resolution and rapid data acquisition^14,21–28^. This recent emergence of high data-rate single-photon LiDAR systems, which employ SPAD detectors, has stimulated research into potential new applications that seem well-suited to this detector technology such as autonomous navigation, environmental monitoring, and subsea mapping. SPAD-based LiDAR systems have been successfully demonstrated in several challenging scenarios including long-range depth imaging^29–34^, imaging through clutter^35,36^, non-line-of-sight detection of targets hidden from view^37–39^, and imaging of targets in high levels of scattering media^27,40–45^.

Although CMOS-based SPAD arrays offer advantages in terms of large format detector arrays, they are typically restricted to wavelengths of less than 1000 nm. The use of short-wave infrared (SWIR) wavelengths in LiDAR systems can have several advantages over shorter wavelengths, such as reduced in-band solar background^46^, higher atmospheric transmission in clear conditions^47,48^, and improved transmission in some obscurants when compared to near-infrared wavelengths^36^. Most importantly, being outside the retinal hazard region (400–1400 nm)^49^, the selection of 1550 nm as the operating wavelength enables the use of a higher average optical power illumination beam whilst remaining eye-safe when compared to visible and near-infrared bands. The increased optical power levels afforded by use of the SWIR region can result in a greater maximum attainable LiDAR range and/or improvement in achievable depth resolution for shorter acquisition times.

The use of more conventional imaging approaches for “seeing” through degraded visual environments, such as those caused by natural and man-made obscurants (e.g., dust, fog, smoke, and haze), pose an impediment to situational awareness in scenarios such as airborne navigation, surveillance and reconnaissance^50–52^. Previously, sensing technologies that have been used in the presence of obscurants include RADAR based sensors^53,54^, and passive sensors based on thermal imaging^55,56^. There has been some previous work on using LiDAR for imaging through obscurants, but this was mostly laboratory-based work or range-gated LiDAR techniques such as Burst Illumination LiDAR (BIL) systems^43,44^. The single-photon sensitivity and excellent depth resolution of TCSPC LiDAR systems have offered potential for high-resolution 3D imaging through atmospheric obscurants^41,42^. This paper reports the imaging through approximately 10 m of obscurant, of moving targets over distances of 50 and 150 m using full-field single-photon detection.

In recent years, there has been great interest in the implementation of image processing algorithms designed to reconstruct images from sparse photon data, and several algorithms have demonstrated good performance for data obtained in free-space scenarios where the return signal is very low and the background is relatively high^29,57–64^. Currently, a major bottleneck in the use of single-photon LiDAR systems is that these algorithms typically suffer from the disadvantage of long execution times (generally 10 s to 100 s of seconds), limiting their use in applications that rely on near instantaneous target analysis. Real-time reconstruction of 3D scenes from single-photon data was achieved recently using highly scalable computational tools being run on a graphics processing unit (GPU)^65^. However, this algorithm is not optimized for imaging through turbid media which contain high and non-uniform background levels, but instead was specifically designed for complex scenes typically containing more than one surface in each pixel. This paper presents a new approach for the real-time processing of single-photon data acquired from imaging scenes through obscurants, which can deal with particularly high and non-uniform background levels. The proposed reconstruction algorithm combines ingredients from recent state-of-the-art algorithms including the use of an advanced statistical modelling^48,53,54^, the exploitation of spatio-temporal information, and combining non-linear parameter estimation and filtering steps^66,67^ to deliver a robust estimation strategy. The resulting algorithm allows scene reconstruction from extremely noisy data with a non-uniform temporal (or depth) profile, as expected from propagation through high levels of obscurants, whilst quantifying the uncertainty of the depth and intensity reconstruction, which is essential for practical 3D imaging applications. This algorithm includes a new statistical formulation that exploits the multi-scale and multi-temporal information of single-photon LiDAR data to improve robustness to noise and quantify the uncertainty of the estimates. In addition, this algorithm allows the use of latent variables that can be updated in parallel, improving computational costs. Finally, this model produces simple iterations that can be efficiently implemented.

In this paper we present an active imaging system based on the single-photon ToF approach to obtain depth and intensity profiles of moving targets through high levels of obscurant. The bistatic system comprised a pulsed laser source with an operational wavelength of 1550 nm and a maximum average optical output power level of 220 mW, and an InGaAs/InP SPAD detector array which is highly efficient in the SWIR region.

The combination of this active imaging system with the proposed advanced algorithm allowed, for the first time, the reconstruction of depth and intensity profiles of static and moving targets, placed in high levels of atmospheric obscurant at stand-off distances of up to 150 m in both indoor and outdoor environments.

## Results

### Experiment layout

The LiDAR transceiver was arranged in a bistatic optical configuration, and used a 32 × 32 InGaAs/InP SPAD detector array. A pulsed fibre laser source operating at a wavelength of 1550 nm was used to flood-illuminate the scene using an average optical power of 220 mW at a pulse repetition rate of 150 kHz. The overall instrumental jitter was 485 ps full width at half maximum. The choice of 1550 nm wavelength operation meant that the transceiver system used in these measurements was characterised as being eye-safe at all distances between the system and target, i.e., the system had a nominal ocular hazard distance of zero metres. Further details of the transceiver are described in “Methods” below and in Supplementary Material 2.

**Figure 1:**
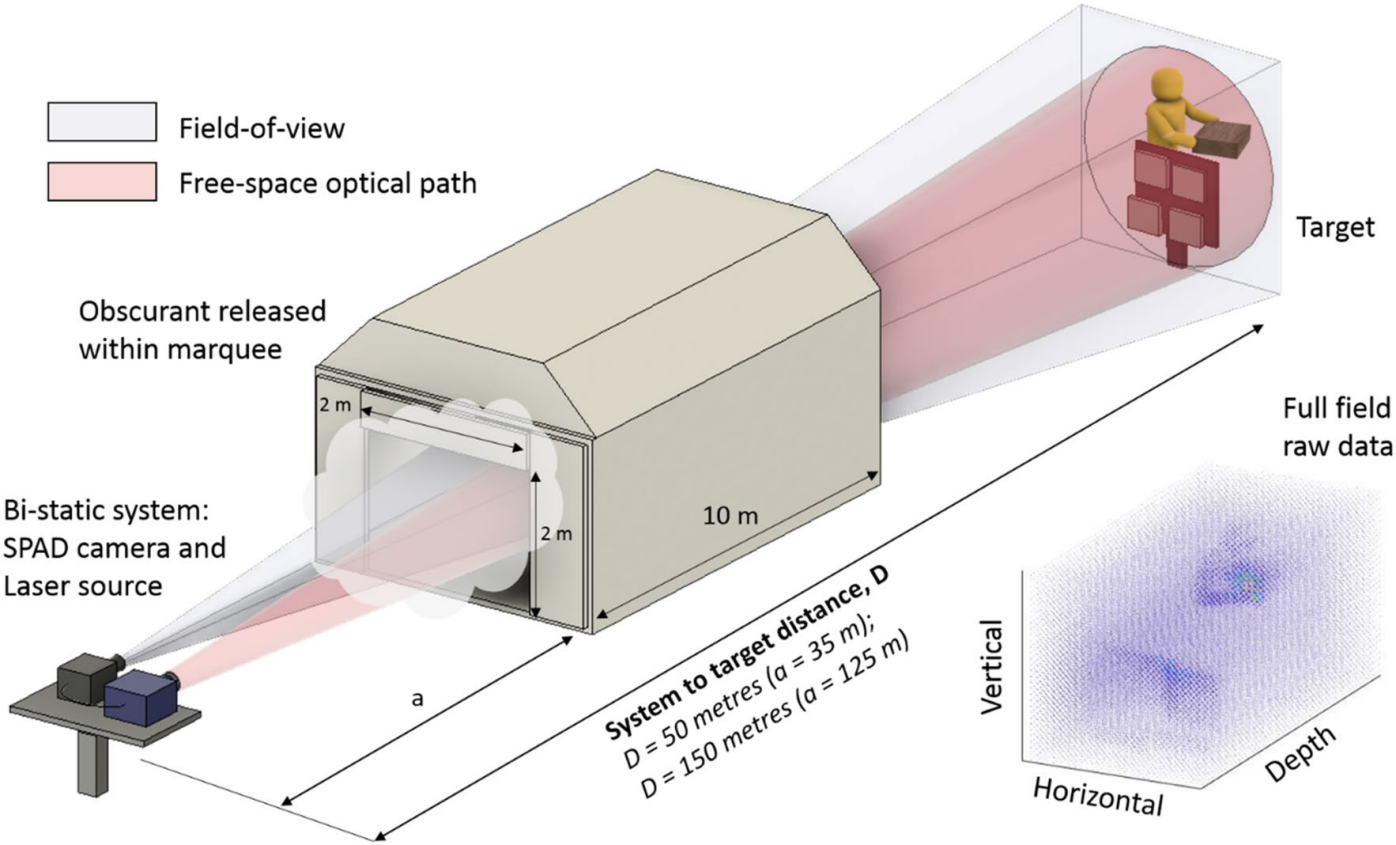
Measurement set-up for the bistatic time-of-flight depth imaging system comprising a 32 × 32 format InGaAs/InP SPAD detector array and a pulsed fibre laser source. The time-of-flight approach was used to obtain depth information of targets at ranges of 50 and 150 m from the transceiver location, through various densities of an oil-based vapour (contained inside a 10-m-long marquee). The marquee was positioned at distances of approximately 35 m and 125 m from the LiDAR system for the for 50 m and 150 m ranges, respectively. An example of a raw data cube representing detected photon counts is also shown. The data was obtained by imaging a moving man located behind fixed boards through an obscurant. The raw data shown was acquired for 100 ms, which is composed of 15,000 binary frames. During the indoor 50 m measurements the fog dispersed backwards and enveloped the target area.

**Figure 2:**
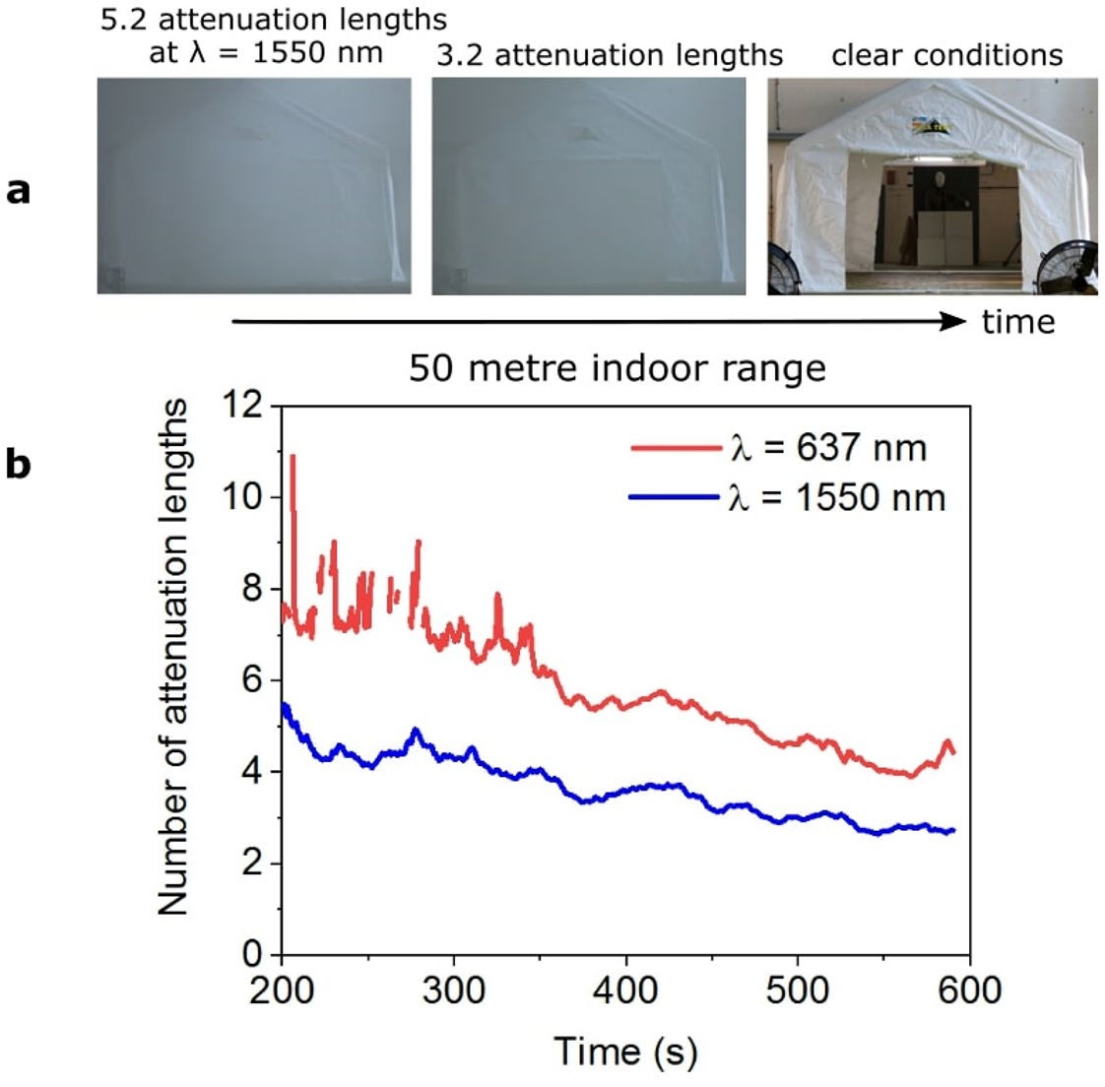
(**a**) Photographs of the target scene taken in visible light with a compact digital camera as the obscurant disperses during a measurement set. Over several minutes, the obscurant disperses and the attenuation between the LiDAR transceiver and the target reduces. The corresponding number of attenuation lengths at λ = 1550 nm between transceiver and target is given for each image. (**b**) A comparison of the number of attenuation lengths as a function of time over the duration of a measurement set for wavelengths of 1550 nm and 637 nm. The λ = 1550 nm measurements were obtained using the bistatic depth imaging system at a stand-off distance of 50 m.

Measurements were performed both indoors and outdoors in daylight for stand-off ranges of 50 m and 150 m respectively with the setup as shown schematically in Fig. 1. For each measurement, a smoke gun was used to produce an oil-based vapour with droplet sizes on the order of a few microns. In order to contain the obscurant, it was released in a polyethylene tent or marquee with dimensions of 3 m (H) × 4 m (W) × 10 m (L), with 2 m × 2 m openings at either end. This was positioned in the line-of-sight between the system and target area and was located at distances of approximately 35 m and 125 m from the LiDAR system for the for 50 m and 150 m ranges, respectively. For each measurement, the closed tent was filled with the oil vapour until a sufficient density was achieved. The tent doors were then opened and measurements of the target scene were made while the oil vapour slowly dispersed (see Fig. 2a). For the indoor range, two large fans were used to help control the dispersal of the obscurant for improved homogeneity. It is worth noting that during the indoor measurements at a range of 50 m the obscurant dispersed throughout the building, enveloping the target area. A depth calibration was performed using a target consisting of four flat 500 × 500 mm wooden panels separated at 100 mm increments in the direction of the beam path. These four flat surfaces were placed immediately adjacent to each other, as shown in Fig. 1 and the transceiver was aligned to incorporate all four target surfaces within the field of view. The target surfaces were painted matt white, which resulted in uniform scattering of the incident illumination, and were used for depth and reflectance calibration measurements. This 3D wooden panel target was included in the scene and used to evaluate the system performance when imaging through different densities of the oil vapour.

### Estimation of attenuation

The number of attenuation lengths between the system and the target at the operating wavelength of 1550 nm was calculated from the number of photon counts collected in clear conditions (*n*_*0*_) and the number of photons collected through obscurant (*n)*, from the same area on the 3D wooden panel target, under otherwise identical operating conditions. The attenuation coefficient (*α*) for the level of obscurant present in the chamber, and hence the number of attenuation lengths (N_AL_), for the one-way distance (*d*) between the transceiver and target, was then calculated from the Beer-Lambert law^68^ as follows:

1 $$N_{AL} = \alpha d = \frac{1}{2}\ln \left[ {\frac{{n_{0} }}{n}} \right]$$

where, due to the low return signal through high levels of the obscurant, *n* and *n*_*0*_ were calculated from the photon counts summed over a 9 × 9 pixel neighbourhood for a 1 s data acquisition in order to acquire an accurate measurement of the number of attenuation lengths.

A transmissometer was also used to perform a second independent measurement of the number of attenuation lengths, in the visible region of the spectrum, corresponding to the level of obscurant. The transmissometer operated at a wavelength of λ = 637 nm and was set up to one side of the LiDAR system location, approximately 10 m closer to the target. The transmissometer laser beam was directed through the marquee to a corner cube retro-reflector (located near the target position) and reflected back to a fibre-coupled Si photodiode. The transmissometer measurements were synchronised with those of the LiDAR system but it is important to note that the two systems did not follow the exact same optical path, although care was taken that the transmissometer data were as representative as possible of the optical attenuation experienced by the bistatic LiDAR system. A comparison of the number of attenuation lengths over the duration of a measurement set for wavelengths of 1550 nm and 637 nm is shown in Fig. 2b. This figure clearly demonstrates the advantage of λ = 1550 nm operation for this type of obscurant, with significantly less attenuation compared with the visible wavelength shown.

### Observation model

3D imaging through obscurants raises several challenges due to back-scattered photons leading to high and non-uniform background level in the observed histograms. This limits the use of the classical matched filter (or cross-correlation) strategy for depth estimation and increases the requirement for statistical confidence guarantees regarding the reconstructed scene. In addition, for 3D video representation, the system acquires successive data cubes, highlighting the need for an online robust processing approach to account for temporal data correlations while dealing with the high volume of acquired data. In the following, we describe the observation model and proposed reconstruction algorithm, which offers a solution to previous challenges, i.e., it allows robust and online processing of 3D LiDAR imaging data through obscurants while providing uncertainty estimates of the depth value.

The TCSPC system provides $K$ successive data cubes composed of two spatial dimensions (i.e., pixel locations) and one time-of-flight dimension (related to depth). The *k*th cube/frame is denoted by ${y}_{t,n,k}$ and contains histograms of photon counts at pixel location, $n \in \left\{1, \dots , N\right\}$, time-of-flight bin $t \in \left\{1, \dots , T\right\}$ and cube number $k \in \left\{1, \dots , K\right\}$. Figure 1 shows an example of a raw data cube.

**Figure 3:**
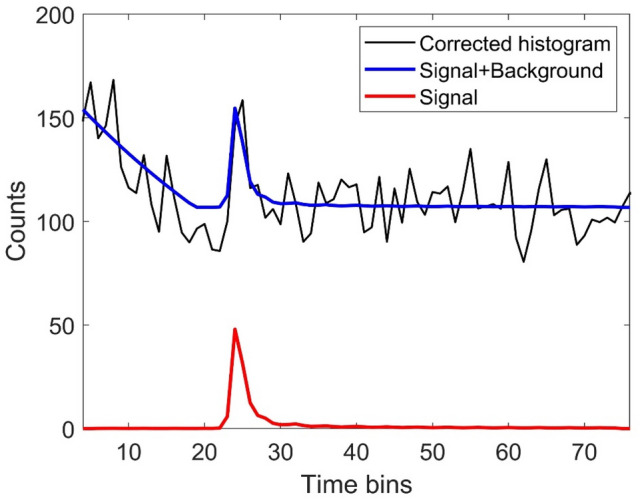
An example of a timing histogram from an individual pixel taken from a flat area of the 3D depth chart at 50 m range through 4.5 attenuation lengths of obscurant, showing the observed histogram after correction as discussed in the main text (black line), the estimated signal plus background (blue), and the estimated target signal (red). This histogram contains 80 timing bins due to the use of a 20 ns gate duration by the detector. Further details in main text and Fig. 4.

Assuming at most one surface per-pixel, each photon count can be assumed to be drawn from the Poisson distribution $P\left(.\right)$ as follows^57,61^:

2 $$y_{t,n,k} \sim P\left[ {r_{n,k} f\left( {t - d_{n,k} } \right) + b_{t,n,k} } \right],$$

where $f$ represents the system impulse response assumed to be known from a calibration step, ${d}_{n,k}>0, {r}_{n,k}>0$ denote the distance from the sensor and reflectivity of the object for the $k$ th data, ${b}_{t,n,k}>0$ gathers the background and dark counts of the detector. To account for obscurants, several studies showed that the background level might vary with respect to the depth observation window such as described by Satat et al.^41^**,** which approximated the noise using a Gamma shaped distribution. In this paper, we assume the signal is located in the decreasing tail of the background distribution leading to the approximation ${b}_{t,n,k} = max({a}_{n,k}{exp}^{-{c}_{n,k}t} ,{\tilde{e }}_{n,k})$**,** where ${a}_{n,k}$ and ${c}_{n,k}$ respectively represent the amplitude and decreasing rate of the exponential, and ${\tilde{e }}_{n,k}$ is a constant background level per-pixel. The observation model is finally given by (see Fig. 3 for an example of a real timing histogram obtained by the system using a gate duration of 20 ns (i.e., 80 timing bins)).

3 $${y}_{t,n,k} \sim P\left[{r}_{n,k}f\left(t-{d}_{n,k} \right) + max({a}_{n,k}{exp}^{-{c}_{n,k}t} ,{\tilde{e }}_{n,k})\right].$$

Our goal is to adaptively and robustly estimate the vector $\Theta =({\Theta }_{s }, {\Theta }_{n})$ that includes the target $\Theta =(D,R)$ and noise ${\Theta }_{n}=(A,C,\tilde{E })$ parameters using the successively observed histograms ${y}_{t,n,k}, \forall t, n, k$, and exploiting their statistics in Eq. (3).

### Reconstruction algorithm

The proposed solution deals with previous challenges by adopting a three-step strategy. The first pre-processing step is used for the estimation of noise parameters using an efficient median estimator for the constant level, ${\tilde{e }}_{n,k}$, and approximate analytical estimators for the exponential parameters. This step allows the approximate unmixing of signal and background counts and can be easily adapted to different background distributions to deal with different scenarios.

The second step represents the core of the algorithm and aims at the robust estimation of depth and reflectivity images by adopting a hierarchical Bayesian model. This approach introduces prior distributions for the unknown parameters to account for their known properties, i.e., positivity, and multiscale/multitemporal correlations. The combination of the measurement statistics summarised in the likelihood and the parameters prior distributions leads to a posterior distribution on the parameters. The latter summarises the probability information regarding each parameter allowing the extraction of its estimate and the quantification of its uncertainty. A main contribution relies on the appropriate choice of prior that accounts for the parameter properties while leading to fast parameter estimates. Multiscale information will be considered as it accounts for spatial correlations between pixels and has shown its importance in many restoration algorithms^57,62^ especially in extreme conditions due to a sparse-photon regime or high noise levels. For added robustness, the proposed algorithm also accounts for multi-temporal information by considering previously estimated depth and reflectivity frames to restore the current frame. These priors are accounted for through the introduction of a depth latent variable denoted **x** that decouples the multiscale spatial and multitemporal information of the depth allowing parallel and fast parameter estimation. To preserve edges between distinct surfaces, we adopt a Laplace prior for **x** as it promotes depth sparsity through the implicit $\ell_{1}$-norm^69^, that has demonstrated good results for several depth reconstruction applications^70^.

The reflectivity is assumed spatially smooth and this is introduced by exploiting multiscale information. Depth uncertainty is represented using a variance parameter for **x**, which is assigned a conjugate non-informative scale prior. The resulting posterior distribution is exploited by considering maximum a-posteriori estimators for the parameters. The latter are obtained using a coordinate descent algorithm that iteratively maximize the parameters conditional distributions. The resulting algorithm alternates between robust non-linear parameter estimation (efficient weighted median^71^) and a filtering step (generalised soft-thresholding), which are commonly observed steps in several state-of-the-art algorithms^65–67^ and optimisation algorithms^72^.

The third optional step relates to data super-resolution to improve the spatial quality of the images. Inspired from the depth maximum a-posteriori estimate, super-resolution is performed using a combination of a weighted median operator with a point cloud filtering step.

**Figure 4:**
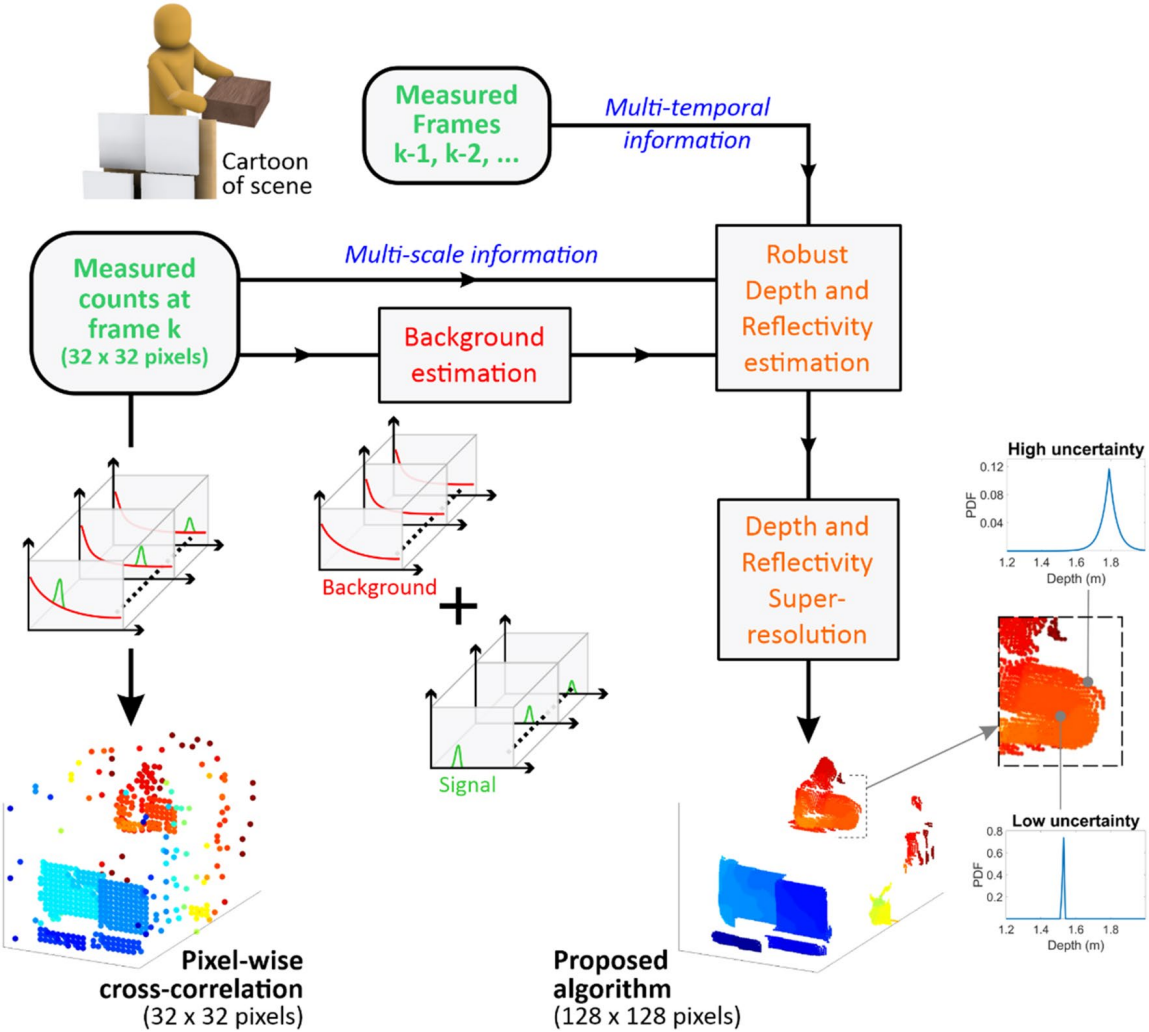
Description of the M2R3D algorithm. The algorithm first estimates the background level (as shown in red). Then the depth and reflectivity are estimated by a robust statistical procedure accounting for multiscale and multi-temporal information. The algorithm estimates a depth statistical distribution for each pixel (denoted PDF for probability density function). The overall procedure is rapid, enabling > 10 frames per second using Matlab.

The main steps of the proposed algorithm, named Median-based Multi-scale Restoration of 3D images (M2R3D), are summarised in Fig. 4 and are described with additional detail in the Supplementary Information. The acquired histograms have non-uniform timing bins due to timing issues with the detector array read-out circuitry (see section I-D of the Supplementary Information for further information). This effect is corrected before applying the proposed strategy and the correcting procedure is denoted as ‘histogram corrections’ in this paper.

### Depth and intensity reconstruction at 50 m range

**Figure 5:**
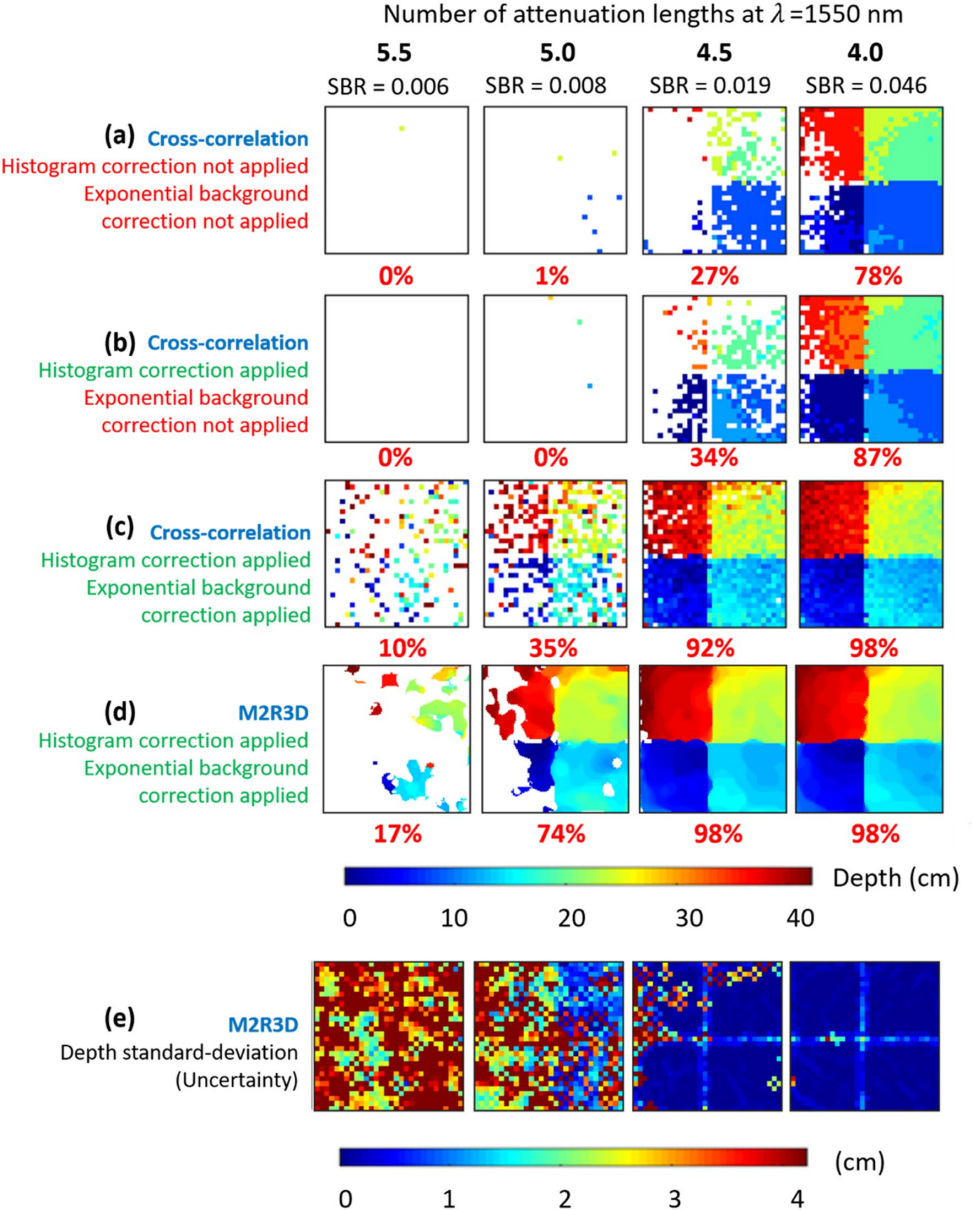
Depth profiles and uncertainty maps of the 3D panel target at a stand-off distance of 50 m indoors for various level of attenuation. The data acquisition time was 1 s. The profiles were reconstructed using the cross-correlation with no corrections applied to the histogram or exponential background (row (**a**)), corrections to the histogram only (row (**b**)), and with corrections applied to both the histogram and the exponential background (row (**c**)). Row (**d**) shows the high-resolution depth profiles (128 × 128 pixels) reconstructed using the M2R3D algorithm with a processing time of ~ 90 ms per frame in Matlab (details in main text). A true positive percentage value for a depth absolute error (DAE) of 5.6 cm is shown in red below each reconstruction (see main text and Supplementary Information). Row (**e**) illustrates the uncertainty of the depth estimate obtained from the M2R3D algorithm, shown as the standard deviation of the estimated depth statistical distribution. The SBR is also shown for each attenuation length (details in main text).

A series of measurements were performed indoors using a scene containing the 3D wooden panel target positioned at a range of 50 m (100 m round-trip) from the system. The results were reconstructed using both a simple pixel-wise cross-correlation algorithm (i.e., matched filter of the raw histogram with the impulse response of the system) and the proposed M2R3D algorithm. The 3D panel chart was flood-illuminated with 220 mW average optical output power from the laser source. The field of view (FoV) of the SPAD detector array camera setup was approximately 0.53 × 0.53 m meaning that only the central region of the 1 × 1 m target was imaged at this range. Attenuation results for this measurement obtained by the transmissometer (for λ = 637 nm) and the depth imaging system (for λ = 1550 nm) are shown in Fig. 2b. The attenuation results are shown from 200 s until the end of the measurement (590 s) since the transmissometer was unable to obtain reliable attenuation values at the very high densities of obscurant observed at the beginning of a measurement cycle. These results indicate that λ = 1550 nm light has considerably lower attenuation than that at λ = 637 nm demonstrating a clear benefit in the use of SWIR illumination over shorter illumination wavelengths for this type of obscurant. Figure 5 shows reconstructed depth profiles of the target at the stand-off distance of 50 m with varying obscurant density corresponding to 4.0, 4.5, 5.0 and 5.5 attenuation lengths between the LiDAR transceiver and target, measured at the illumination wavelength of 1550 nm. The data was acquired over a time of 1 s in each case, at an average optical output power level of 220 mW. As mentioned previously regarding measurements on the 50 m range, the obscurant dispersed throughout the target area and fully submerged the target. In order to investigate the consequence of obscurant density on the reconstruction quality we investigate several scenarios as indicated in the rows of Fig. 5. In rows from top to bottom: (a) the cross-correlation was first applied to non-corrected histograms; (b) the cross-correlation was applied to corrected histograms; (c) the cross-correlation was applied to corrected histograms while accounting for an exponential background; and finally (d) the M2R3D algorithm was used to reconstruct depth profiles and (e) the depth uncertainty, as quantified by the standard-deviation of the depth conditional distribution. Note that the M2R3D algorithm provides super-resolved depth profiles composed of 128 × 128 pixels. A depth threshold was set such that all depths outside a pre-determined distance around the target (in this case 0.6 m) were considered to be inaccurate estimates, disregarded, and presented in the reconstructed depth profiles as empty pixels for clarity (shown as white pixels in Fig. 5). In addition, the signal-background-ratio (SBR) estimated from M2R3D is given for each corresponding attenuation length. SBR is defined as the ratio of the average signal photons-per-pixel (S_ppp_) and the average background photons-per-pixel (B_ppp_). More details on these parameters are given in the supplementary material.

The results in Fig. 5 show that the 3D panel target can only be partially reconstructed using the cross-correlation algorithm at up to 4.5 attenuation lengths with no histogram corrections nor background consideration (Fig. 5a), and similarly with only histogram correction (Fig. 5b). When both histogram correction and an exponential background are considered, using the cross-correlation provides a better reconstruction at 4.5 attenuation lengths, and a partial reconstruction can be achieved for obscurant densities up to 5.0 attenuation lengths (Fig. 5c). However, using the M2R3D algorithm (Fig. 5d), the 3D panel target can be partially reconstructed at 5.0 attenuation lengths and fully reconstructed at 4.5 attenuation lengths. In fact, a partial reconstruction was also made using the M2R3D algorithm at up to 5.5 attenuation lengths. Significantly, M2R3D also quantifies depth uncertainty as represented in (Fig. 5e) by showing the standard deviation of the estimated depth statistical distribution. It can be seen that higher uncertainty is observed around object edges and in the presence of high obscurant densities. In particular, standard deviations (of a Laplace distribution) greater than 4 cm are observed for badly estimated pixels at greater than 5.0 attenuation lengths.

True positive percentage values, which represent the percentage of pixels satisfying a given depth within a given error^61^, were calculated for the results obtained with each algorithm are shown in red in Fig. 5. As an example, for the 5.0 attenuation lengths results the true positive percentage values satisfying a depth absolute error (DAE) of 5.6 cm are 1%, 0%, 35%, and 75% for each algorithm respectively, illustrating a significant quantitative improvement in the reconstructed depth profiles by using both the exponential background and the proposed M2R3D algorithm. To compute the DAE, a 32 × 32 pixel reference depth map was generated using data obtained in the absence of fog. These values were calculated using the lower resolution depth maps due to the unavailability of a high-resolution ground truth. A more detailed description of the true positive percentage, DAE, and further analysis are supplied in the Supplementary Information. We also provide video 1 which displays the results of M2R3D and cross-correlation algorithm (with all corrections) when processing this data at different time instances.

### Depth and intensity reconstruction at outdoor 150 m range

**Figure 6:**
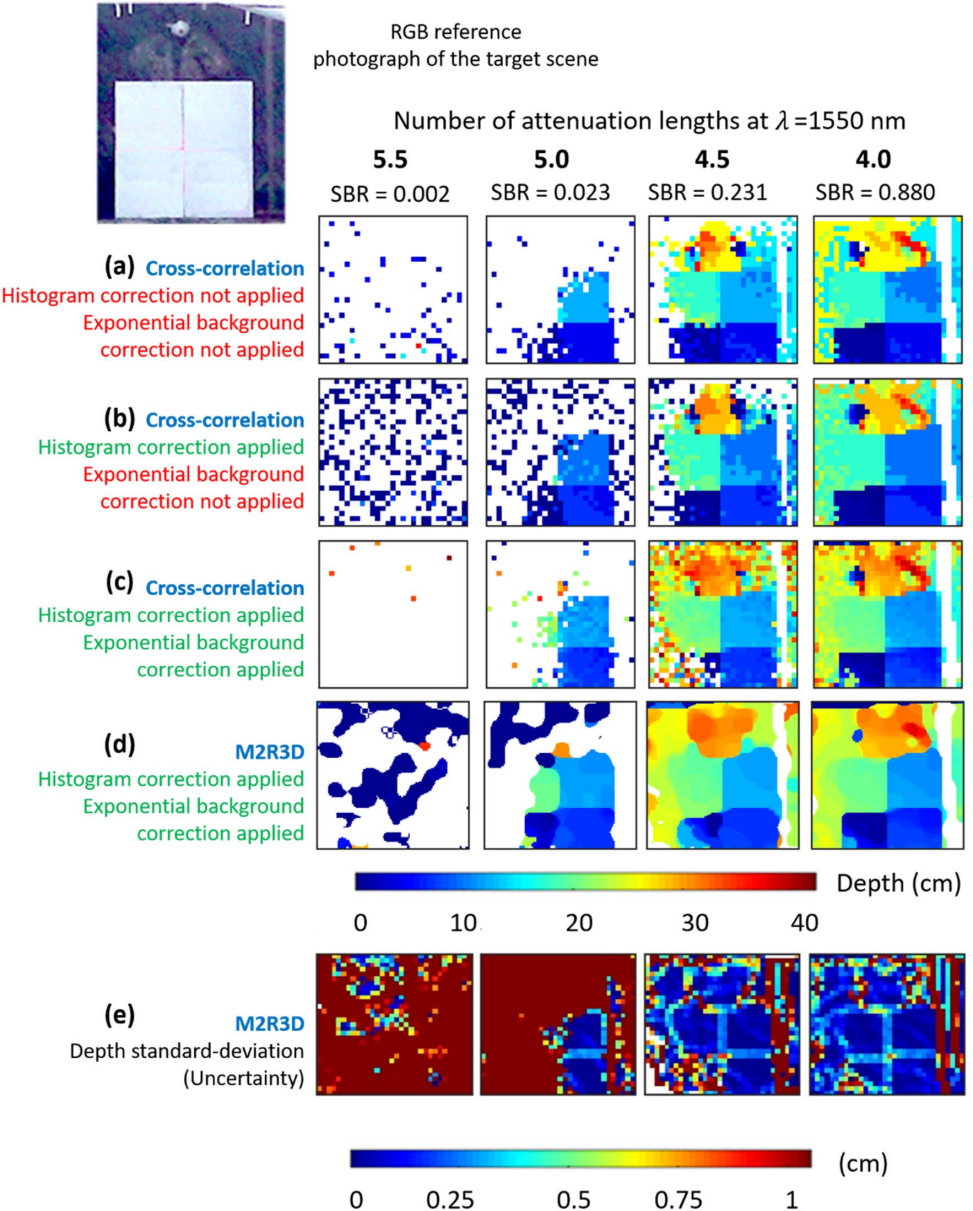
Depth profiles and uncertainty maps of the 3D wooden panel target and actor in various densities of obscurant at a stand-off distance of 150 m outdoors using a maximum average optical power level of 220 mW and a data acquisition time of 1 s. The depth profiles were reconstructed using the cross-correlation with no corrections applied to the histogram or exponential background (row (**a**)), corrections to the histogram only (row (**b**)), and with corrections applied to both the histogram and the exponential background (row (**c**)). Row (**d**) the super-resolved depth profiles (128 × 128 pixels) reconstructed using the M2R3D algorithm with a processing time of ~ 90 ms per frame (details in main text). Row (**e**) illustrates the uncertainty of the depth estimate obtained from the M2R3D algorithm, shown as the standard deviation of the estimated depth statistical distribution. An RGB photograph of the scene with no obscurant present is shown for reference. The SBR is also shown for each attenuation length.

A second set of measurements was performed outdoors in daylight conditions at a range of 150 m (300 m round-trip) from the transceiver location. The 3D wooden panel target was housed within an intermodal container to help shield it from adverse weather conditions. An actor was also situated directly behind the 3D wooden panel target. Since the horizontal and vertical FoV was three times larger at 150 m than at 50 m, the resultant data contained information from the entire 3D wooden panel target, the actor, as well as superfluous information from the back wall of the container. As with the 50 m range measurements shown above, the depth profiles of the scene were reconstructed using cross-correlation with no corrections applied, with only the histogram corrections applied, with both histogram corrections and an exponential background, and with the proposed M2R3D algorithm, as shown in Fig. 6.

The results shown in Fig. 6a–d indicate that a partial depth reconstruction can be made at up to 5.0 attenuation lengths using both cross-correlation and the proposed M2R3D algorithm at a stand-off distance of 150 m. It should however be noted that a high depth uncertainty is observed for most regions at attenuation lengths of greater or equal to 5.0 as highlighted in Fig. 6e. A full reconstruction of the target scene could be made at 4.5 attenuation lengths for both the cross-correlation with both histogram and exponential background correction (Fig. 6c) and the M2R3D algorithm (Fig. 6d). In this case, the depth profile reconstructed using corrected cross-correlation is noisier than that of the M2R3D algorithm. However, the reconstruction using the M2R3D algorithm does tend to over-smooth the fine target details, such as the arms and face of the actor, while assigning higher depth uncertainty levels for these regions. These results demonstrate the potential of the system for rapid three-dimensional imaging outdoors in high levels of obscurants, in high levels of solar background, and adverse weather conditions using SWIR wavelengths. Since the human target was moving in this measurement set, no ground truth could be obtained and therefore no true positive percentage values could be calculated for this data.

### Real-time processing of moving 3D scenes in obscurant

**Figure 7:**
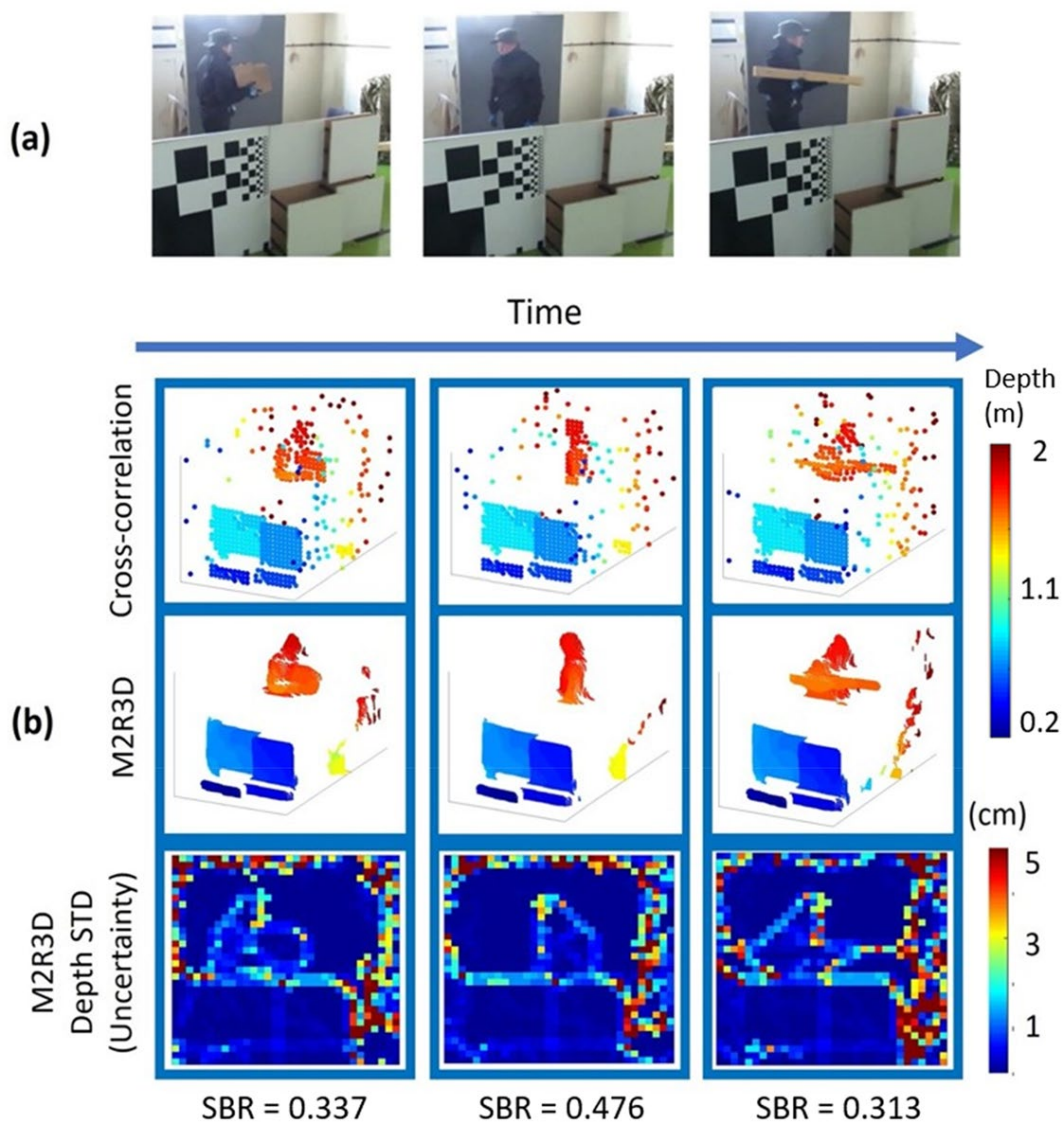
(**a**) RGB reference photographs taken of the moving scene with no obscurant present, using a visible camera. (**b**) Stationary frames extracted from video of estimated depth profiles and uncertainty maps of the moving target scene measured through the oil-based vapour obscurant. These frames were reconstructed using the cross-correlation algorithm with histogram correction (top) and the proposed M2R3D algorithm (middle and bottom). In this scene (left) a man is carrying a box, (middle) the man is empty handed, and (right) the man is carrying a plank of wood. At this point there was approximately 3 attenuation lengths at λ = 1550 nm between the system and the target, and each still frame represents 100 ms of data acquisition and ~ 90 ms of data processing (details in main text). The corresponding SBR is also shown for each of the frames.

This section describes a further set of measurements using a moving target at a distance of 50 m through the oil-based vapour obscurant. The target scene included the 3D wooden panel target and an actor walking from side to side through the scene holding alternating objects (a box and a plank of wood). The measurements were acquired using the same system configuration and parameters as previously described. For these measurements 15,000 successive binary frames, each with an acquisition time of approximately 6.7 µs, were aggregated to create a 10 frames per second video with an overall duration of several minutes. Figure 7 shows three stationary depth profiles obtained with cross-correlation (with histogram corrections and assuming no background), and the proposed M2R3D algorithm. These stationary frames from the video correspond to the point in time when there were approximately 3 attenuation lengths at λ = 1550 nm between the system and the target.

These results highlight the benefit of the M2R3D algorithm in removing false detections and improving spatial resolution. As expected, higher depth uncertainty is estimated for pixels located at object edges, around small object features (e.g., the man’s hat) or in regions where the pixels of the SPAD array camera demonstrate lower single-photon detection efficiency (e.g., the region at the right in the depth map). The processing time of the M2R3D algorithm is around ~ 90 ms per frame enabling real time processing (latency lower than one frame). Video 2 shows the results of the M2R3D algorithm and the cross-correlation algorithm (with all corrections) when processing this data at different time instances. Other data and comparison algorithms have also been considered in the supplementary materials, which shows the advantages of the proposed algorithm.

## Discussion

This paper presents depth and intensity profiling of static and moving targets in high levels of obscurant at stand-off ranges of 50 m and 150 m outdoors using the time-correlated single-photon counting technique. The eye-safe time-of-flight imaging system comprised a 32 × 32 InGaAs/InP SPAD detector array and a picosecond pulsed fibre laser with an illumination wavelength of 1550 nm and a maximum average optical output power of 220 mW. This was used to perform three-dimensional imaging at up to 5 attenuation lengths for a range of 50 m and 4.5 attenuation lengths at 150 m. A comparison of light propagation through the oil-based vapour (i.e., artificial fog) was also made between short-wave infrared wavelengths and visible wavelengths, illustrating a significant wavelength dependence in the attenuation due to increased scatter at the shorter wavelengths with this particular obscurant, as seen in our previous work^42^. Of course, wide scale implementation of this approach means that attention must also be paid to the development of cost-effective LIDAR sources, since eye-safe implementation in the SWIR region permits much higher optical powers than possible in the near-infrared.

This paper presented an advanced image processing algorithm, which is capable of real-time reconstruction of depth and intensity profiles of moving targets in high levels of obscurants. In contrast to other algorithms^57,60,65^, the M2R3D uses an observation model that accounts for the presence of obscurants which typically can exhibit a non-uniform depth profile, allowing a robust reconstruction of the scene while providing uncertainty estimates of the depth measurements using multiscale and multi-temporal information. In the Supplementary Materials, it is shown that the proposed M2R3D algorithm performs better than several state-of-the-art single-photon depth and intensity reconstruction algorithms^57,60,65^ especially in the presence of high or non-uniform background levels, which are often observed in presence of obscurants^41^. The real-time processing allowed the demonstration of depth imaging of moving targets, a critical attribute necessary for future implementations on mobile platforms. A key aspect of this algorithm is that the processing time is not proportional to the number of photon events in a histogram, which is particularly important for the specific case of imaging in obscurants which necessarily incurs an unusually high level of back-scattered photon events from the illumination. More details and comparison with other algorithms are given in Supplementary Information.

The results presented in this paper demonstrate the potential for the implementation of single-photon counting approaches using InGaAs/InP SPAD detector arrays in modern embedded systems (e.g., driverless cars). While the high timing resolution, long-range capabilities, and low optical power levels inherent to the TCSPC approach fulfil many of the requirements of current systems, further investigation is necessary to evaluate the system and proposed algorithm in more challenging scenarios. Therefore, future work will include measurements made in natural fog environments and high levels of precipitation, and the investigation of targets travelling at higher velocities at longer ranges. Generalising the proposed model to account for alternative background profiles, and to account for pixels without target information are also important points that will be investigated. Also, the use of parallel processing tools and graphical processing units (GPU) will be investigated to decrease frame processing times to a duration of a few milliseconds in order to utilise next generation, larger format detector arrays. We have demonstrated depth image reconstruction using a LiDAR system with a limited optical field of view, and must acknowledge that many applications will require an extended FoV requiring optical scanning in at least one dimension, which will, in turn, reduce pixel dwell time. Future work will concentrate on the implementation of a scanning strategy and examining the trade-offs between FoV, spatial resolution, and dwell time.

## Methods

### System description

The LiDAR system was based on an InGaAs/InP 32 × 32 SPAD detector array camera manufactured by Princeton Lightwave. The pixel elements of the detector array were on a 100 μm square pitch, resulting in the active area of the sensor having dimensions of ~ 3.2 mm × 3.2 mm. This sensor can be used for single-photon and low-light detection in the SWIR wavelength range of 1400–1620 nm. The sensor was operated at a frame rate of 150 kHz, and this provided the synchronised trigger for the pulsed laser source. The SPAD detector array was configured to use a 250 ps timing bin width (the smallest available), which corresponds to a 3.75 cm depth resolution. For the measurements reported here, a 20 ns gate duration was selected, which corresponds to a total of 80 histogram bins and is equivalent to a measurement depth range of 3 m. A 300 mm effective focal length objective lens operating at f/3.5 was attached to the sensor unit to collect photons scattered back from the target scene and resulted in a FoV of approximately 11 mrad horizontally and vertically. This means that the sensor imaged an area of approximately 0.53 × 0.53 m at a stand-off distance of 50 m, and 1.6 × 1.6 m at a stand-off distance of 150 m, which corresponded to each individual pixel covering an area of approximately 16 × 16 mm and 50 × 50 mm, respectively.

The LiDAR system was implemented using a bistatic configuration and a schematic diagram of the setup is included in Supplementary Information Fig. 10. The illumination channel (to which the laser source was fibre-coupled) and SPAD detector array camera channel were mounted side by side. The illumination source was a pulsed fibre laser with a central operating wavelength of 1550 nm, and run at a repetition rate of 150 kHz (clocked by the camera control electronics), which resulted in a pulse-width of 413 ps, and a maximum average optical output power of approximately 220 mW. Further details on the system set-up can be found in Supplementary Material 2.

### Algorithm implementation

The algorithm performs weighted median filtering using the C ++ efficient implementation proposed in^71^. All other steps were implemented using MATLAB R2019a on a computer with an Intel(R) Core(TM) i7-4790 CPU running at 3.60 GHz and with 32 GB RAM.

## Supplementary Information

Supplementary Information 1.

Supplementary Video 1.

Supplementary Video 2.

## Data Availability

The data will be made available upon publication of this work. Algorithm available on request.
