# Supplementary material for: Robust real-time 3D imaging of moving scenes through atmospheric obscurant using single-photon LiDAR
Source: Sci Rep. 2021 May 27;11:11236. doi: 10.1038/s41598-021-90587-8 (PMC8159934; doi:10.1038/s41598-021-90587-8)
Supplement: Supplementary file 1 — Supplementary Information 1. [file 41598_2021_90587_MOESM1_ESM.pdf]

Robust real-time 3D imaging of moving scenes through atmospheric obscurant using single-photon LiDAR

Rachael Tobin, Abderrahim Halimi, Aongus McCarthy, Philip J. Soan, Gerald S. Buller

Supplementary material 1: Algorithm's description

This section introduces the proposed statistical algorithm for the reconstruction of 3D images of moving targets, acquired using single-photon LiDAR through high densities of atmospheric obscurants. The algorithm has three main objectives: (i) robust processing of Lidar data obtained in presence of a high level of noise, (ii) fast processing allowing online processing of successive 3D frames, (iii) deliver uncertainty quantification of the obtained estimates. To reach those goals, the advanced algorithm adopts a statistical Bayesian formulation with carefully chosen likelihood and prior distributions. Due to the proposed formulation, the estimates and their uncertainty measures are obtained using an iterative algorithm with efficient updates allowing online data processing. Results on simulated and real data show more robustness to noise and faster results than state-of-the-art algorithms. The next section describes the problem formulation. The proposed Bayesian model is presented in Section II, followed by the description of its estimation algorithm in Section III. Sections IV, and V finally compare the proposed approach to state-of-the-art algorithms on simulated and real data.

I. Problem formulation

This section introduces the statistical observation models that relate the measured tagged-photons to the parameters of interest $\boldsymbol{\Theta} = (\boldsymbol{\Theta}^s, \boldsymbol{\Theta}^n)$, which gathers the target $\boldsymbol{\Theta}^s$ and noise $\boldsymbol{\Theta}^n$ parameters.

A. Observation model

The TCSPC system provides time-tagged photon event data that can be accumulated into a timing histogram $\boldsymbol{y}_{n,t}$, denoting the number of detected photons at pixel location $n \in \{1, \cdots, N\}$, and time-of-flight (ToF) bin $t \in \{1, \cdots, T\}$. These histograms (often called cubes of data) contain depth and reflectivity information about the observed target allowing 3D imaging. This process can be repeated at different time instants to acquire 3D videos, in which case the data is denoted $\boldsymbol{y}_{n,t,k}$ where $k \in \{1, \cdots, K\}$ represents the cube's number. Each photon counts can be assumed to be drawn from the Poisson distribution $\mathcal{P}(.)$ as follows [1], [2]:

$$y_{n,t,k} \sim \mathcal{P}\left(s_{n,t,k}\right). \quad (1)$$

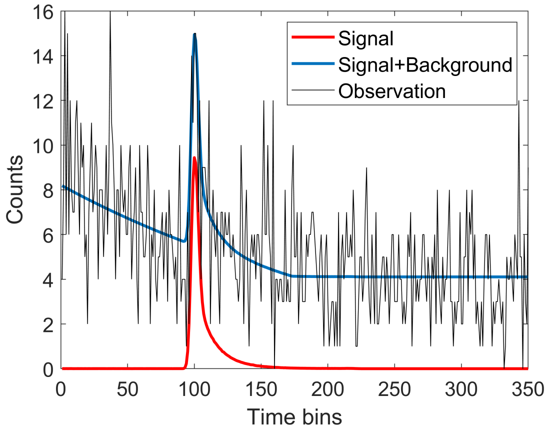

Fig. 1. Example of synthetic data representing a target signal (in red), signal and background (in blue), and an observed histogram (in black) using model (5). For this pixel, the sum of signal counts is 100 counts, and the sum of background counts are 1750 counts leading to an SBR= 0.057.

Assuming the presence of at most one surface per-pixel, the average photon counts can be modelled as a mixture of useful signal, and background counts as follows

$$s_{n,t,k} = r_{n,k} f\left(t - d_{n,k}\right) + b_{n,t,k} \quad (2)$$

where f represents the system impulse response function (IRF) assumed to be known from a calibration step and normalized by its sum (f is obtained using a long integration time, single point measurement on a uniform flat surface in laboratory conditions), $r_{n,k} \geq 0$ is the reflectivity of the object surface for the kth data, $d_{n,k} \geq 0$ denotes the distance of the object from the sensor for the kth data (related to its depth), $b_{n,t,k} \geq 0$ gathers the background and dark counts of the detector (in the following, we will use the "background" term for all events that do not originate from reflections at the target plane). It is assumed that the background $b_{n,t,k}$ depends on t as further explained in next sub-sections.

B. Event based observation model

Model (1) has been used in many studies, and it assumes the availability of histograms of counts. An alternative is to directly model the detected list of photons $z_{n,p,k}$ for the nth pixel and for $p \in \{1, \cdots, \bar{y}_{n,k}\}$, using a mixture of densities as in [1], [3]

$$P(z_{n,p,k}|u_{n,k}, d_{n,k}) = (1-u_{n,k}) g\left(z_{n,p,k}\right) + u_{n,k} f\left(z_{n,p,k} - d_{n,k}\right) \quad (3)$$

where $g(z_{n,p,k})$ denotes the density of background counts, $u_{n,k}$ represents the probability of the detected photon to belong to a target or background and $\bar{y}_{n,k}$ the total number of photons detected in the nth pixel and kth frame. Interestingly, model (3) can be equivalently represented using the following hierarchical model [4]

$$\begin{aligned} P(z_{n,p,k}|h_{n,p,k}, d_{n,k}) &= g(z_{n,p,k})^{1-h_{n,p,k}} f(z_{n,p,k} - d_{n,k})^{h_{p,n,k}} \\ P(h_{n,p,k}|u_{n,k}) &= (1-u_{n,k})^{1-h_{n,p,k}} u_{n,k}^{h_{n,p,k}} \end{aligned} \quad (4$$

where $h_{n,p,k} \in \{0,1\}$ is a latent variable following a Bernoulli distribution of parameter $u_{n,k}$, and taking 1 if the pth photon is reflected from a target and 0 otherwise. Note that marginalizing $h_{n,p,k}$ in this model leads to the mixture model (3). Model (4) decouples the distributions of the photons associated with the target and background, which is particularly interesting for Bayesian approaches as shown later.

C. Background model

In imaging through obscurants or a scattering medium, the background $b_{n,t,k}$ will present high values and might vary with respect to the depth observation window as already observed in [5], [6]. In [6], Satat et al assumed a Gamma shaped background noise based on the multiple scattering of the photon in the obscuring medium. In this paper, we assume that the observation window, and hence the useful signal, is located in the decreasing tail of the background distribution which can be approximated using a decreasing exponential with a minimum background level as follows $b_{n,t,k} = \max\left[a_{n,k}\exp(-c_{n,k}t), \tilde{e}_{n,k}\right]$, where $a_{n,k}$ and $c_{n,k}$ respectively represent the amplitude and decreasing rate of the exponential, and $\tilde{e}_{n,k}$ is a constant level per-pixel. Note here that the rate "$c_{n,k}$" is pixelwise dependent to capture the non-homogeneous variations of the imaging environment. This results in the final model given by (see also Fig. 1)

$$s_{n,t,k} = r_{n,k} f(t - d_{n,k}) + \max\left[a_{n,k}\exp(-c_{n,k}t), \tilde{e}_{n,k}\right]. \quad (5)$$

Our goal is then to adaptively estimate $\boldsymbol{\Theta} = (\boldsymbol{\Theta}^s, \boldsymbol{\Theta}^n)$ that includes the target $\boldsymbol{\Theta}^s = (\boldsymbol{D}, \boldsymbol{R})$ and noise $\boldsymbol{\Theta}^n = \left(\boldsymbol{A}, \boldsymbol{C}, \tilde{\boldsymbol{E}}\right)$ parameters using the observations $y_{n,t,k}$, $z_{n,p,k} \forall n, t, p, k$, while exploiting their statistics in (1) and (4), and the multi-scale/multi-temporal information for robustness to noise.

D. Histogram corrections

The measured histograms exhibit consistent non-uniform timing bins due to issues in the timing read-out circuitry. This effect was also observed and discussed further by Henriksson et al. in section 3.2 of [7]. This leads to a clear periodic fluctuations (with a period of 4 bins) in the measured histograms as shown in Fig. 2 (blue curve) representing the sum of all histograms of an oil-based vapour obscurant scene without target. Assuming the fluctuation pattern is fixed between successive frames, this effect is corrected before applying the proposed strategy and the correcting procedure is denoted as "histogram corrections" in the main article. More precisely, using data with only background counts and no target, we estimate 4 weights/proportions for each successive 4 bins to approximate the fluctuations in the timing bin lengths. We then compensate per-pixel histograms by redistributing the photon counts between bins to approximate equal weights or timing bin lengths. An example of the obtained results is shown in Fig. 2 (red curve) which highlights reduced fluctuations after correction.

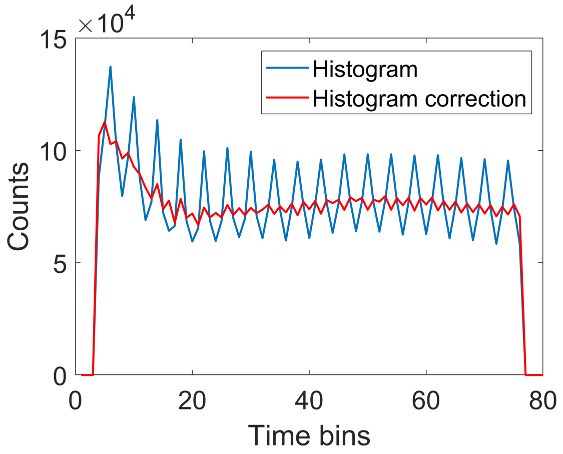

Fig. 2. Example of histogram correction. (In blue) the sum of the measured histograms of all pixels of an oil-based vapour obscurant scene without a target present. (In red) the same sum after applying a per-pixel histogram correction.

II. Hierarchical Bayesian model

The estimation of $\boldsymbol{\Theta}$ is an ill-posed problem due to the high level of noise corrupting the data. In this paper, we adopt a Bayesian approach to alleviate the indeterminacy resulting from ill-posed problems. This approach combines the observation information summarized in the likelihood term, with prior distributions on the unknown parameters to obtain a posterior distribution on the parameters. The latter could be exploited by deriving point estimators and additional measures of uncertainty about the estimates. The following sub-sections introduce the proposed Bayesian model.

A. Likelihood

1) Approximate Likelihood: Assuming independence between the observed photon ToFs leads to the following likelihood for the nth pixel of the kth frame

$$P(\boldsymbol{z}_{n,k}|\boldsymbol{h}_{n,k}, d_{n,k}) = \prod_{p=1}^{\bar{y}_n} g(z_{n,p,k})^{1-h_{n,p,k}} f(z_{n,p,k} - d_{n,k})^{h_{n,p,k}} \quad (6)$$

where $\bar{y}_n$ represents the number of photons in the nth pixel. It is common to approximate the system IRF with a Gaussian function [8], [9], in which case the likelihood in (6) becomes

$$P(\boldsymbol{z}_{n,k}|\boldsymbol{h}_{n,k}, d_{n,k}) = \prod_{p=1}^{\bar{y}_n} g(z_{n,p,k})^{1-h_{n,p,k}} \mathcal{N}(z_{n,p,k}; d_{n,k}, \sigma^2) \quad (7)$$

where $\mathcal{N}(x; \mu, \sigma^2)$ denotes the Gaussian distribution of x with expectation μ and variance σ^2. Assuming the availability of

an estimator $\hat{h}_{n,p,k}$ of $h_{n,p,k}$ which allows the separation of signal and background counts leads to

$$P(\boldsymbol{z}_{n,k}|\hat{\boldsymbol{h}}_{n,k}, d_n) = \left[\prod_{p=1}^{\bar{b}_n} g\left({}^b z_{n,p,k}\right)\right] Q\left({}^s\boldsymbol{z}_{n,k}\right) \times \mathcal{N}(d_{n,k}; \bar{d}_n, \frac{\sigma^2}{\bar{s}_{n,k}}) \quad (8)$$

where $Q\left({}^s\boldsymbol{z}_{n,k}\right)$ is a function of signal counts, $\bar{d}_{n,k} = \frac{\sum_{p=1}^{\bar{s}_n} {}^s z_{n,p,k}}{\bar{s}_n}$, ${}^s z$ and ${}^b z$ represent the photons reflected from a target and background counts, respectively, and $\bar{s}_{n,k}$ and $\bar{b}_{n,k}$ denote their number in the nth pixel. The resulting likelihood highlights a Gaussian distribution for the depth variable $d_{n,k}$. This approximate likelihood for $d_{n,k}$ can also be obtained by considering the Poisson model (1) for signal photons (no-background) leading to

$$P({}^s\boldsymbol{y}_{t,k}|r_{n,k}, d_{n,k}) \propto \mathcal{G}\left(r_{n,k}; 1+\bar{s}_{n,k}, 1\right) \bar{Q}\left({}^s\boldsymbol{y}_{t,k}\right) \times \mathcal{N}(d_{n,k}; \bar{d}_{n,k}, \frac{\sigma^2}{\bar{s}_{n,k}}) \quad (9)$$

where ${}^s\boldsymbol{y}_{t,k}$ represent the histogram of signal counts, $\propto$ stands for proportional to, and $\mathcal{G}(x;.,.)$ denotes the gamma distribution with shape and scale parameters. The formulation (9) is interesting for the estimation of the reflectivity $r_{n,k}, \forall n, k$. Note that the simple depth maximum likelihood estimator (MLE) reduces to a log-matched filtering of the histogram with the impulse response of the system when assuming the absence of noise. The MLE approach is often used because of its simplicity, however, it obviously yields poor results from data acquired in the presence of obscurants as it does not consider spatial correlations between pixels, hence the need for an advanced robust method.

2) Multiscale information: It is common to use the multiscale information available within the histograms to improve the performance of maximum likelihood estimation for Lidar data [1], [10]–[12]. The key observation is that spatially downsampled histograms, which are still Poisson distributed, lead to depth and reflectivity estimates with lower noise at a price of a reduced spatial resolution. In this paper, we adopt a similar strategy by considering L downsampled version of the histogram of counts. Considering predefined L graphs of neighbours $\phi^{1,\cdots,L}$, spatially downsampled version of the histograms $\boldsymbol{Y}$ can be obtained for each time instant k leading to $\boldsymbol{Y}_k^{\ell}, \ell \in \{1, \cdots, L\}$. Assuming independence between these histograms lead to L likelihood distributions for photon events

$$P\left(\boldsymbol{z}_{n,k}^{(\ell)}|\hat{\boldsymbol{h}}_{n,k}^{(\ell)}, d_{n,k}^{(\ell)}\right) = \left[\prod_{p=1}^{\bar{b}_n^{(\ell)}} g\left({}^b z_{n,p,k}^{(\ell)}\right)\right] Q\left({}^s\boldsymbol{z}_{n,k}^{(\ell)}\right) \times \mathcal{N}\left(d_{n,k}^{(\ell)}; \bar{d}_n^{(\ell)}, \frac{\sigma^2}{\bar{s}_n^{(\ell)}}\right), \quad (10)$$

$\forall \ell \in 1, \cdots, L$, where $\ell = 1$ is the original cube, and $\ell > 1$ is a downsampling index, e.g., 3×3 for $\ell = 2$, 5×5 for $\ell = 3$, etc. Similarly, one can express the likelihood of histograms as follows

$$P(\boldsymbol{y}_{n,k}^{(\ell)}|r_{n,k}^{(\ell)}, d_{n,k}^{(\ell)}) \propto \mathcal{G}\left(r_{n,k}^{(\ell)}; 1+\bar{s}_{n,k}^{(\ell)}, 1\right) \bar{Q}\left(\boldsymbol{y}_{n,k}^{(\ell)}\right) \times \mathcal{N}(d_{n,k}^{(\ell)}; \bar{d}_{n,k}^{(\ell)}, \frac{\sigma^2}{\bar{s}_{n,k}^{(\ell)}}). \quad (11)$$

B. Prior distribution for depth

The depth parameters are assigned priors enforcing spatial smoothness. This is achieved by introducing a latent variable $\boldsymbol{x}$ that is related to all multi-scale depth parameters. More precisely, we consider a mixture of Gaussian conditional prior distributions for $\boldsymbol{x}$ as follows

$$x_{n,k}|d_{n,k}^{(1,\cdots,L)}, x_{\nu_n,\psi_k}, \gamma_{\nu_n,n}^{(1,\cdots,L)}, \eta_{\nu_n,n}^{(\psi_k)}, \epsilon_n \sim \prod_{n' \in \nu_n}\left[\prod_{\ell=1}^{L} \mathcal{N}\left(x_{n,k}; d_{n',k}^{(\ell)}, \gamma_{n',n}^{(\ell)}\epsilon_n^2\right)\right] \times \left[\prod_{k' \in \psi_k} \mathcal{N}\left(x_{n,k}; x_{n',k'}, \eta_{n',n}^{(k')}\epsilon_n^2\right)\right] \quad (12)$$

where ν_n (resp. ψ_k) denotes the spatial (resp. temporal) neighbourhood of the nth pixel (resp. kth frame), $d_{n',k}$ denotes the mean, $\gamma_{n',n}^{(\ell)} \geq 0$, $\eta_{n',n}^{(k')} \geq 0$ are weights for the variance, and $\epsilon_n > 0$ is the variance of $\boldsymbol{x}_n$. Model (12) shows that the latent variable $\boldsymbol{x}$ links to the L depth variables (as indicated in Fig. 3) providing a robust approximation of the true depth map by considering correlations between pixels. This model also shows a temporal correlation between successive latent variables $x_{k,k-1,\ldots}$ through the variable $\boldsymbol{v}$. It should be noted that (12) does not enforce positivity on the depth parameter $\boldsymbol{x}$, however, this will be ensured as indicated Section III-B.

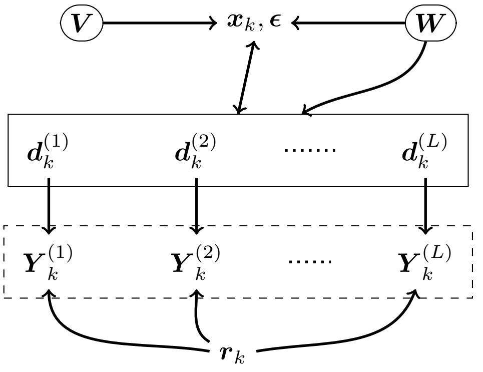

Fig. 3. DAG for the observations, parameter and hyperparameters of the marginalized model. The user fixed parameters appear in circles, the observations in a dashed box and the rectangular box gathers the joint multi-scale depth parameters.

C. Prior distribution for reflectivity

Similarly to depth, spatial smoothness could be enforced to reflectivity by considering latent variables (for example using the gamma Markov random field prior [13]. In this work, the multi-scale estimates $r_{n,k}^{(\ell)}$ implicitly contain spatial

correlations allowing the definition of a simple prior. More precisely, we assume that $r^{(1)}_{n,k} = \cdots = r^{(L)}_{n,k} = r_{n,k}$ as this will lead to fast estimation for reflectivity as detailed later.

D. Hyperparameter priors

While the distribution in (12) introduces spatial correlations between pixels through the latent variable $\boldsymbol{x}$, it will lead to over smoothed depth edges due to the implicit l_2-norm in the Gaussian distribution, i.e., it will join distinct depth surfaces. To alleviate this issue and conserve edges, a better prior distribution should consider sparse promoting norms such as l_1. To obtain such effect, we consider an exponential prior distribution for the hyper-parameters $\gamma^{(\ell)}_{n',n}, \eta^{(k')}_{n',n}$ as follows

$$\begin{aligned} \gamma^{(\ell)}_{n',n} &\sim \mathcal{E}\left(\gamma^{(\ell)}_{n',n}; w^{(\ell)}_{n',n}\right) \\ \eta^{(k')}_{n',n} &\sim \mathcal{E}\left(\eta^{(k')}_{n',n}; v^{(k')}_{n',n}\right) \end{aligned} \tag{13}$$

where $\mathcal{E}(.;.)$ denotes the exponential distribution with the fixed parameters $w^{(\ell)}_{n',n} \geq 0$, $v^{(k')}_{n',n} \geq 0$ that satisfy $\sum_{\ell,n'} w^{(\ell)}_{n',n} + \sum_{k',n'} v^{(k')}_{n',n} = 1$ and should be carefully selected. The combination of (13) and (12) will provide the desired edge preserving effect as indicated in the next section.

The parameter $\epsilon_n, \forall n$ should be positive. Assuming prior independence between the parameters $\epsilon_n, \forall n$ and in absence of additional information, the non-informative scale prior is chosen for this parameter as follows

$$f(\epsilon_1, \cdots, \epsilon_N) = \prod_{n=1}^{N} \frac{1}{\epsilon_n} \mathbb{I}_{\mathbb{R}^+}(\epsilon_n) \tag{14}$$

where $\mathbb{I}_{\mathbb{R}^+}(.)$ denotes the indicator function on $\mathbb{R}^+$.

E. Marginalized posterior distribution

The proposed Bayesian model is summarized in the directed acyclic graph (DAG) displayed in Fig. 3. The joint posterior distribution of this Bayesian model can be computed from the following hierarchical structure (after dropping indices for clarity)

$$\begin{aligned} f(\boldsymbol{X}, \boldsymbol{D}, \boldsymbol{R}, \boldsymbol{\epsilon}|\boldsymbol{Y}) \propto f(\boldsymbol{Y}|\boldsymbol{R}, \boldsymbol{D}) f(\boldsymbol{D}, \boldsymbol{X}|\boldsymbol{\Gamma}, \boldsymbol{\eta}, \boldsymbol{\epsilon}) \\ f(\boldsymbol{\Gamma}|\boldsymbol{W}) f(\boldsymbol{\eta}|\boldsymbol{V}) f(\boldsymbol{\epsilon}). \end{aligned} \tag{15}$$

Equations (15) and (11) clearly highlight the independence of the depth related parameters $\boldsymbol{D}, \boldsymbol{X}, \boldsymbol{\Gamma}, \boldsymbol{W}$ and the reflectivity ones $\boldsymbol{R}, \boldsymbol{\eta}, \boldsymbol{V}$. Akin to the Bayesian lasso model [14], [15], the parameters $\boldsymbol{\Gamma}, \boldsymbol{\eta}$ can be marginalized leading to Laplace distributions instead of the Gaussian distributions as shown in (17). Note that the marginalization reduces the number of parameters to estimate while making the estimation procedure more robust. The marginalized posterior distribution is finally given by

$$\begin{aligned} f(\boldsymbol{X}, \boldsymbol{D}, \boldsymbol{R}, \boldsymbol{\epsilon}|\boldsymbol{Y}) \propto f(\boldsymbol{Y}|\boldsymbol{R}, \boldsymbol{D}) \\ f(\boldsymbol{D}, \boldsymbol{X}|\boldsymbol{W}, \boldsymbol{V}, \boldsymbol{\epsilon}) f(\boldsymbol{\epsilon}). \end{aligned} \tag{16}$$

This distribution contains complete information regarding the parameters of interest $\boldsymbol{X}, \boldsymbol{D}, \boldsymbol{R}, \boldsymbol{\epsilon}$ and their uncertainties. A common approach is to extract point estimators such as the minimum mean square estimator (MMSE) or maximum a-posteriori (MAP) estimator. In this paper, we consider the marginalized MAP estimator of all parameters, while uncertainty regarding depth is summarized in the parameter $\epsilon_n, \forall n$.

III. Estimation algorithm

This section describes a coordinate descent algorithm to approximate the maximum of the marginalized posterior distribution in (16) with respect to the parameters $\boldsymbol{X}, \boldsymbol{D}, \boldsymbol{R}, \boldsymbol{\epsilon}$ [16], [17]. The iterative algorithm sequentially updates a parameter of interest while holding the other parameters fixed. This is equivalent to sequentially maximizing the conditional distributions associated with each parameter. This process is repeated until the algorithm has converged to a local minimum of the negative log-posterior. The algorithm's main steps are presented in Algo. 1 and described with more details in the following sections. It is worth noting that the resulting algorithm alternates between robust non-linear parameter estimation (line 10) and a filtering step (line 11), which are commonly observed steps in several state-of-the-art algorithms [18]–[20] and optimization algorithms [21].

Algorithm 1 Estimation algorithm

1: Input:
2: $\boldsymbol{Y}_k, \boldsymbol{x}_{\psi_k}, L, \phi^{1,\cdots,L}, \boldsymbol{\Theta}^n(k)$
3: Generate low resolution data:
4: Generate low-resolution histograms $\boldsymbol{Y}^{(\ell)}_k, \ell \in \{1, \cdots, L\}$ using $\phi^{1,\cdots,L}$
5: Estimate $\bar{\boldsymbol{d}}^{(\ell)}, \bar{\boldsymbol{r}}^{(\ell)}$ from $\boldsymbol{Y}^{(\ell)}$ using $\boldsymbol{\Theta}^n(k)$, as in (26)
6: Compute the weights $\boldsymbol{W}, \boldsymbol{V}$ as in (27), (30)
7: Coordinate descent algorithm
8: Estimate $\boldsymbol{r}_k$ using analytical mode in (22)
9: **while** conv$=0$ **do**
10: Update $x_{n,k}, \forall n, k$ using WMF in (18)
11: Update $\boldsymbol{d}^{(\ell)}, \forall \ell$ using threshold operator in (19)
12: Update $\boldsymbol{\epsilon}$ using analytical mode in (21)
13: Set conv$=1$ if the convergence criteria are satisfied
14: **end while**
15: Optional: data super-resolution
16: Compute $\boldsymbol{d}^{\text{HR}}_k, \boldsymbol{r}^{\text{HR}}_k$ as in Algo. 2
17: Output:
18: $\boldsymbol{x}_k, \boldsymbol{r}_k, \boldsymbol{\epsilon}$ (optional: $\boldsymbol{d}^{\text{HR}}_k, \boldsymbol{r}^{\text{HR}}_k$)

A. Updating $\boldsymbol{X}$

The parameters of $\boldsymbol{X}$ are independent allowing parallel updating of $x_{n,k}, \forall n$. The conditional distribution of each $x_{n,k}, \forall n, k$ is given by

$$\begin{aligned} x_{n,k}|d^{(1,\cdots,L)}_{n,k}, x_{\nu_n,\psi_k}, w^{(1,\cdots,L)}_{\nu_n,n}, v^{(\psi_k)}_{\nu_n,n}, \epsilon_n \sim \\ \prod_{n' \in \nu_n} \left[\prod_{\ell=1}^{L} \mathcal{L}\left(x_{n,k}; d^{(\ell)}_{n',k}, \frac{\epsilon_n}{\sqrt{2 w^{(\ell)}_{n',n}}} \right) \right] \\ \times \left[\prod_{k' \in \psi_k} \mathcal{L}\left(x_{n,k}; x_{n',k'}, \frac{\epsilon_n}{\sqrt{2 v^{(k')}_{n',n}}} \right) \right] \end{aligned} \tag{17}$$

where $\mathcal{L}(.;.,.)$ denotes Laplace distribution. Maximizing this distribution is equivalent to

$$\begin{aligned} \hat{x}_{n,k} &= \underset{x}{\operatorname{argmin}}\, \mathcal{C}(x), \quad \text{with} \\ \mathcal{C}(x) &= \sum_{n' \in \nu_n} \left[\sum_{\ell=1}^{L} \sqrt{w_{n',n}^{(\ell)}} |x - d_{n',k}^{(\ell)}| \right. \\ &+ \left. \sum_{k' \in \psi_k} \sqrt{v_{n',n}^{(k')}} |x - x_{n',k'}| \right]. \end{aligned} \tag{18}$$

This is the equation of a weighted median filter (WMF) which has several efficient implementations (e,g, [22]). Note that the solution of (18) will be positive provided that $x_{n',k'} > 0$ and $d_{n',k}^{(\ell)} > 0$, which is ensured during initialization.

B. Updating $\boldsymbol{D}$

The variables $d_1^{(\ell)}, \cdots, d_N^{(\ell)}$ are independent and spatial correlation is introduced through the latent variable $\boldsymbol{x}$. This is interesting as it allows the parallel implementation of $d_n^{(\ell)}, \forall n$ with respect to n and ℓ. Straightforward computations show that the update $d_n^{(\ell)}$ is given by

$$\begin{aligned} d_n^{(\ell)} &= \underset{d}{\operatorname{argmin}} \left[\frac{\bar{s}_{n,k}^{(\ell)} ||d - \bar{d}_n^{(\ell)}||^2}{2\sigma^2} \right. \\ &+ \left. \sum_{n' \in \nu_n} \frac{\sqrt{2 w_{n',n}^{(\ell)}}}{\epsilon_{n'}} |d - x_{n',k}| \right]. \end{aligned} \tag{19}$$

This is a generalization of the well known soft-threshold operator which can be efficiently solved. Note that the solution of (19) will be positive provided that $x_{n',k} > 0$ and $\bar{d}_n^{(\ell)} > 0$ which is ensured during initialization.

C. Updating depth variance: $\boldsymbol{\epsilon}$

The conditional distribution of ϵ_n is an inverse-gamma distribution given by

$$\epsilon_n | \boldsymbol{X}, \boldsymbol{D}, \boldsymbol{W}, \boldsymbol{V} \sim \mathcal{IG}\left[L + \bar{K} + \bar{N}, \mathcal{C}\left(x_{n,k}\right)\right] \tag{20}$$

whose mode is given by

$$\hat{\epsilon}_n = \frac{\mathcal{C}\left(x_{n,k}\right)}{L + \bar{K} + \bar{N} + 1} \tag{21}$$

where $\bar{K}$ (resp. $\bar{N}$) is the number of temporal (resp. spatial) neighbours.

D. Updating reflectivity parameters: $\boldsymbol{R}$

The updates of reflectivity parameters are independent from depth parameters, allowing parallel computations. The maximum likelihood estimator of $r_{n,k}$ is given by

$$\hat{r}_{n,k} = \frac{\sum_{\ell=1}^{L} r_{n,k}^{(\ell)}}{L}, \forall n. \tag{22}$$

E. Background estimation

Our algorithm assumes known coefficients $\boldsymbol{h}_{n,k}, \forall n, k$, which can be deduced from known noise parameters as indicated in the input of Algo. 1. Model (5) approximates the noise using three parameters $\boldsymbol{\Theta}_{n,k} = (\tilde{e}_{n,k}, a_{n,k}, c_{n,k})$ where $\tilde{e}_{n,k}$ represents a constant minimum noise level, $a_{n,k}$ its amplitude and $c_{n,k}$ its slope. These parameters will be approximately estimated as described below, where the focus is to obtain analytical estimators suitable for fast computations.

1) Estimation of $\tilde{e}_{n,k}$*:* The maximum likelihood estimate of $\tilde{e}_{n,k}$ in absence of a target ($r_{n,k} = 0$) is given by $\tilde{e}_{n,k} = \sum_{t=1}^{T} \frac{y_{n,t,k}}{T}$, which is the average of the detected counts of each pixel. However, in presence of a target component and an exponential decay of the background, the model assumes each histogram (i.e., pixel) has a constant level plus a narrow signal peak due to a narrow system impulse response. Under these considerations, the parameter of interest is the background level while the signal peak is seen as an outlier. Therefore, our solution considers the median of the detected counts as an efficient and robust approximate estimate of $\tilde{e}_{n,k}$, i.e.,

$$\hat{e}_{n,k} = \text{median}(\boldsymbol{y}_{n,:,k}). \tag{23}$$

2) Estimation of $a_{n,k}$*:* An approximate estimator can be obtained by analysing the case of a target absence ($r_{n,k} = 0$) and no background minimum level ($\tilde{e}_{n,k} = 0$). In this case, the maximum likelihood estimator of the parameter is given by $a_{n,k} = \frac{\sum_{t=1}^{T} y_{n,t,k}}{\sum_{t=1}^{T} \exp(-c_{n,k} t)}$, which is a normalized averaging of the pixel counts. To alleviate the dependence on the unknown parameter $c_{n,k}$ (and thus avoid iterative steps), one can approximate this expression using a point-wise estimator. For this, note that $y_{n,t,k} = a_{n,k} \exp(-c_{n,k} t)$ is an exponentially decreasing function, reaching its maximum for $\hat{a}_{n,k} = \max(y_{n,t,k}), \forall t$. The latter equation will be considered as our approximate estimator for $\hat{a}_{n,k}$, while only considering the 20 first histograms bins after assuming no target is located at these first time bins.

3) Estimation of $c_{n,k}$*:* Given the previously estimated value of $\tilde{e}_{n,k}$, one can easily select the observed counts in the range interval $t \in [1, \min(20, T_1)]$, where T_1 represents the intersection of the decreasing observed counts with the constant background level $\tilde{e}_{n,k}$, after assuming the absence of target for $t < 20$ (i.e., $d_{n,k} > 20$ bins). For this interval, the log-likelihood of $c_{n,k}$ (with the other parameters fixed to their point estimates) can be expressed as

$$\mathcal{C}(c_{n,k}) = \sum_{t=1}^{\min(20,T_1)} [-\hat{a}_{n,k} \exp(-c_{n,k} t) - y_{n,t,k} c_{n,k} t]. \tag{24}$$

An analytical expression of the estimate is obtained after using a second order Fourier series approximation of the exponential function, leading to

$$\hat{c}_{n,k} = \frac{\sum_{t=1}^{\min(20,T_1)} t(\hat{a}_{n,k} - y_{n,t,k})}{\sum_{t=1}^{\min(20,T_1)} t^2 \hat{a}_{n,k}}. \tag{25}$$

4) Estimation of $\boldsymbol{h}_{n,k}$*:* At this stage, the noise parameters $\boldsymbol{\Theta}^n = \left(\boldsymbol{A}, \boldsymbol{C}, \tilde{\boldsymbol{E}}\right)$ are estimated and will help unmix signal

from background counts. More precisely, the signal counts are first located using a log-matched filter strategy as follows

$$\hat{d}^{(\ell)}_{n,k} = \mathrm{argmax}_{d_{n,k}} \sum_{t=1}^{T} \log[f(d_{n,k}-t)] \max(0, y^{(\ell)}_{n,t,k} - q^{(\ell)} \hat{b}_{n,t,k}).$$

The signal counts are then defined as

$$y^{s,(\ell)}_{n,t,k} = \max(y^{(\ell)}_{n,t,k} - q^{(\ell)} \hat{b}_{n,t,k},\, 0), \forall n$$

for $t \in [t_l, t_h]$; where $t_l = \max(1, \hat{d}^{\ell}_{n,k} - 3\sigma)$, $t_h = \min(T, \hat{d}^{\ell}_{n,k} + 3\sigma)$, σ represents the standard deviation of the Gaussian system impulse response and $q^{(\ell)}$ represents the number of neighbours in ϕ^{ℓ}. Depth and reflectivity maximum likelihood estimates can then be obtained using (11) leading to

$$\bar{r}^{(\ell)}_{n,k} = \bar{s}^{(\ell)}_{n} = \sum_{t=t_l}^{t_h} y^{s,(\ell)}_{n,t,k}, \text{ and } \bar{d}^{(\ell)}_{n,k} = \frac{\sum_{p=1}^{\bar{s}^{(\ell)}_n} z^{s,(\ell)}_{n,p,k}}{\bar{s}^{(\ell)}_n}. \quad (26)$$

F. Weights selection

The choice of the weights is very important and will have a direct impact on the algorithm performance. Several strategies have been considered in the literature where the choice can be based on the spatial distance between points, similarity of their values, etc [23]–[25]. In this paper, we define the weights based on multi-scale and multi-temporal information.

Inspired by [26] which adopted the rank order mean approach to unmix signal from background counts, our selection of the multi-scale weights $\boldsymbol{W}$ is based on the empirical observation that the depth map at a given scale $\bar{\boldsymbol{d}}^{(\ell)}$ should be close to its median depth image $\underline{\boldsymbol{d}}^{(\ell)}$ (with a graph of neighbours ϕ^0, for example 3×3 neighbours in a uniform grid) and the median of the following scale $\underline{\boldsymbol{d}}^{(\ell+1)}$. Armed with this observation, we assign low weights for pixels that differ significantly from their corresponding pixels in $\underline{\boldsymbol{d}}^{(\ell)}$ and $\underline{\boldsymbol{d}}^{(\ell+1)}$, i.e.,

$$\begin{aligned} w^{(\ell)}_{n,n'} &= w_{\text{norm}} \left[\prod_{\ell'=1}^{\ell-1} \left(1 - w^{(\ell')}_{n,n'}\right)^{\alpha_{\ell'}} \right] \\ &\times \exp\left(- \frac{|\bar{d}^{(\ell)}_n - \underline{d}^{(\ell)}_{n'}|}{\zeta_d \sqrt{q^{(\ell)}}} - \frac{|\bar{d}^{(\ell)}_n - \underline{d}^{(\ell+1)}_{n'}|}{\zeta_d \sqrt{q^{(\ell+1)}}} \right) \end{aligned} \quad (27)$$

for $\ell \in \{1, \cdots, L-1\}$ and

$$\begin{aligned} w^{(L)}_{n,n'} &= w_{\text{norm}} \left[\prod_{\ell'=1}^{L-1} \left(1 - w^{(\ell')}_{n,n'}\right)^{\alpha_{\ell'}} \right] \\ &\times \exp\left(- \frac{|\bar{d}^{(L)}_n - \underline{d}^{(L)}_{n'}|}{\zeta_d \sqrt{q^{(L)}}} \right) \end{aligned} \quad (28)$$

where w_{norm} is a normalization constant, the coefficient ζ_d is easily fixed based on physical considerations related to the impulse response width and it is weighted with the square root of q^{ℓ} to account for the multi-resolution effect. In (27), the product over ℓ' promotes lower scales data if their weights are height. In addition, we define a scale-quality coefficient $\alpha_\ell \in [0,1]$ which describe the overall quality of $\bar{d}^{(\ell)}_n$ as follows

$$\alpha_\ell = \frac{1}{N} \sum_{n=1}^{N} \delta \left(|\bar{d}^{(\ell)}_n - \underline{d}^{(\ell)}_n| \leq \frac{\zeta_d}{q^{(\ell)}} \right) \quad (29)$$

where $\delta(.)$ is the delta function. At scale ℓ, high (resp. low) α_ℓ means that the image is of good (resp. bad) quality and the product in (28) will be activated (resp. deactivated).

Another useful feature of the proposed algorithm when dealing with high background levels is that it can also account for temporal information between frames, which is justified if the speed of the observed object is lower than the frame rate of the system. Note that higher weights $\boldsymbol{V}$ are associated to lower spatial distance between pixels and/or temporal distance between frames, as follows

$$v_{n,n',k'} = \frac{v_{\text{norm}}}{|k-k'| + |x_n - x_{n'}|^2 + |y_n - y_{n'}|^2}, \quad (30)$$

where $\sum_{k',n'} v_{n',k'} = f_c \sum_{l',n'} w^{(\ell)}_{n,n'}$, with $0 < f_c < 1$ a user-fixed parameter representing the confidence level on current frame, $w_{\text{norm}}, h_{\text{norm}}$ are normalization constants ensuring $\sum_{k',n'} v_{n',k'} + \sum_{l',n'} w^{(\ell)}_{n,n'} = 1$. We note finally that the weights could be updated with iterations leading to a pseudo-Bayesian approach [27], but this is out of the scope of this paper and will be left for future work.

G. Stopping criteria

Two criteria are considered to stop the iterative coordinate decent algorithm. The first is maximum number of iterations. The second evaluates the new depth parameter values and stops the algorithm if the following condition is satisfied

$$\left\| \boldsymbol{x}^{(i+1)} - \boldsymbol{x}^{(i)} \right\|_1 \leq \xi \left(\left\| \boldsymbol{x}^{(i)} \right\|_1 + \xi \right). \quad (31)$$

where i denotes the algorithm iterations and ξ is a threshold.

H. Optional postprocessing: data super-resolution

The system acquires data at a frame rate of 150 kHz but at the relatively low spatial resolution of 32×32 pixels. It is therefore necessary to simulate an improved spatial resolution using super-resolution approaches to improve visualisation. This is a well known problem and several methods have been proposed in the literature to deal with it ranging from simple interpolation algorithms (linear, bicubic), fast linear-based algorithms (guided filter [28]), to sophisticated approaches requiring more computational cost as in [29]–[31]. In this paper, we propose a fast algorithm which achieves real-time, good quality reconstruction of 3D scenes. Our approach is based on three successive steps as highlighted in Algo. 2. The first generates crude estimates of the high-resolution images using simple interpolation methods. The second steps improves edge estimation to remove block effects by considering a self-guided weighted-median filtering with a window of size $r \times r$, which can be efficiently implemented using the strategy in [22]. For each pixel, the weights are defined depending on the pixel's similarity to its $r \times r$ neighbours, which filters homogeneous regions while preserving the edges. A final

filtering step is applied to reflectivity using a self guided filter with parameters (radius $= 5, \text{reg} = 10^{-4}$). The depth is smoothed using the following point based-strategy:

$$\boldsymbol{d}^{\text{HR}}_{n,k} = \begin{cases} \frac{1}{q_n} \sum_{n' \in \nu_n} \boldsymbol{d}^{\text{HR}}_{n',k}, \text{ if } \sum_{n' \in \nu_n} \frac{|\boldsymbol{d}^{\text{HR}}_{n,k} - \boldsymbol{d}^{\text{HR}}_{n',k}|}{q_n} \leq \zeta_d/2 \\ \text{median}(\boldsymbol{d}^{\text{HR}}_{\nu_n,k}), \text{ if } \min(|\boldsymbol{d}^{\text{HR}}_{n,k} - \boldsymbol{d}^{\text{HR}}_{n',k}|) > \zeta_d/4 \\ \boldsymbol{d}^{\text{HR}}_{\nu_n,k}, \text{ else.} \end{cases} \tag{32}$$

Algorithm 2 Super-resolution algorithm

Input:
$\boldsymbol{d}^{\text{LR}}_k, \boldsymbol{r}^{\text{LR}}_k$
Initialization:
$\boldsymbol{d}^{\text{HR}}_k$ = nearest-point interpolation of $\boldsymbol{x}_k$
$\boldsymbol{r}^{\text{HR}}_k$ = bicubic interpolation of $\boldsymbol{r}_k$
Refinement of edges:
$\boldsymbol{d}^{\text{HR}}_k$ = self weighted median of $\boldsymbol{d}^{\text{HR}}_k$ with $r = 3$
$\boldsymbol{r}^{\text{HR}}_k$ = self weighted median of $\boldsymbol{r}^{\text{HR}}_k$ with $r = 6$
Smoothing:
$\boldsymbol{d}^{\text{HR}}_k$ = smoothing of $\boldsymbol{d}^{\text{HR}}_k$ as in (32)
$\boldsymbol{r}^{\text{HR}}_k$ = self guided filter of $\boldsymbol{r}^{\text{HR}}_k$ with (radius $= 5$, reg $= 10^{-4}$)
Output:
$\boldsymbol{d}^{\text{HR}}_k, \boldsymbol{r}^{\text{HR}}_k$

IV. Results on Simulated Data

The proposed reconstruction algorithm, called M2R2D, is evaluated and compared to different state-of-the-art algorithms by considering simulated data. More precisely, our algorithm has been compared to the UA [1], Lindell [32]; and NR3D algorithms [12] which are robust to noise, and to the state-of-the-art MANIPOP algorithm [10] that is designed to address multi-peak scenarios. Both UA and MANIPOP algorithms were used with hyper-parameters provided by the authors, except at extreme noise conditions where larger spatial correlations are considered to improve their robustness. The algorithms are compared in terms of depth accuracy using the depth absolute error (DAE) measure DAE$= \frac{1}{N} \left|\left| \boldsymbol{d}^{\text{ref}} - \boldsymbol{x} \right|\right|_1$ where $\boldsymbol{d}^{\text{ref}}$ and $\boldsymbol{x}$ are the reference and estimated depth maps, respectively. The true positive percentage (i.e., percentage of true detections) representing the percentage of pixels satisfying a given depth absolute error (DAE) is also considered. To highlight the benefit of the proposed ℓ_1-based hierarchical Bayesian model, we also compare to the ℓ_2 based model obtained without marginalization, i.e., considering the prior in (12) while fixing $\gamma^{(\ell)}_{n',n} = 1/w^{(\ell)}_{n',n}$ and $\eta^{(k')}_{n',n} = 1/v^{(k')}_{n',n}$. The latter model involves ℓ_2-norms whose edge blurring effect is well known [9], hence the use of the proposed ℓ_1-based regularization. The simulated realistic scenes were obtained from the Middlebury dataset[1] [1], [33], where we considered the bowling scene represented in Fig. 4. We have analysed two scenarios

[1]Available in: http://vision.middlebury.edu/stereo/data/

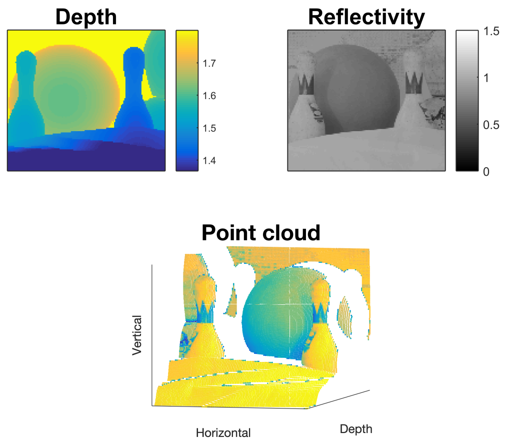

Fig. 4. Synthetic image of a bowling scene. (Top-left) Depth map, (top-right) reflectivity and (bottom) point cloud combining depth and reflectivity. Image from [12].

corresponding to a photon-starved regime in presence of a uniform background, and a high photon return regime in the presence of an exponentially decreasing background due to obscurant. For both scenarios, we analysed different signal-to-background (SBR) levels which are defined as the ratio of the signal and background counts averaged over all pixels [12]. The data of the first scenario has been generated as follows

- We generated histograms of size 123×139 pixels and $T = 300$ bins while considering a real measured impulse response $g(.)$ (having a leading-edge of 10 bins and trailing-edge of 70 bins),
- We considered six levels of target average photons-per-pixel (Sppp for signal ppp, which represents the integrated number of counts in the signal peak after removing background counts) ranging from 0.2 ppp to 25 ppp.
- We considered two average photons-per-pixel background levels (denoted Bppp for background ppp, which represents the per-pixel number of background counts), Bppp $\in \{1, 8\}$ leading to SBR values in the interval $[0.025, 25]$.

This data allows a fair comparison with UA, NR3D and MANIPOP algorithms as they assume a constant background level. Note however that the Lindell algorithm [32] is a learning based algorithm that has been trained on a dataset having different characteristics (e.g., different impulse response width, 1024 time bins, etc). Therefore, to ensure a fair comparison, we used the simulator provided by the authors to simulate data at different Bppp $\in \{2, 10, 50\}$ and Sppp $\in \{2, 5, 10\}$ levels, for the middlebury Art scene of size 185×232 pixels [2]. To highlight the benefit of the proposed algorithm, we have also considered a second set of data having an exponentially decreasing background level as observed on real data. The considered Sppp and Bppp are inspired from those observed on real data (see Table III), as follows

- We generated histograms of size $62 \times 70 \times 340$ while considering a real measured impulse response $g(.)$ (having a

[2]Lindell algorithm is run on a workstation with NVIDIA RTX 6000 GPU as it requires a significant amount of GPU memory.

leading-edge of 10 bins and trailing-edge of 70 bins),

- We considered four levels of target average photons-per-pixel (Sppp) as follows: 100, 250, 500, 1000ppp
- We considered two average photons-per-pixel background levels, Bppp $\in \{1750, 3500\}$ leading to SBR values in the interval $[0.02, 0.57]$, where $a_{n,k} = 2\tilde{e}_{n,k}$, $c_{n,k} = 0.004$ and $t \in [1, 340]$ bins.

In all what follows, the proposed algorithm has been run with the following parameters: $L = 3$ with $q^{(2)} = 3 \times 3$ and $q^{(3)} = 9 \times 9$, $\zeta_d = 3.75$cm, and $f_c = 0.1$. Fig. 5 shows the DAE results obtained by the considered algorithms for the sparse photon regime scenario. The algorithms provided similar performance for both uniform background levels with a clear advantage to M2R3D. As expected, marginalization improves performance as highlighted by comparing M2R3D without marginalization to M2R3D. The proposed strategy showed lower computational cost than UA, NR3D and MANIPOP as indicated in Table I. It should also be noted that both UA and MANIPOP complexity is proportional to Sppp, NR3D scales with the scene complexity (slower to converge for extreme cases), while M2R3D computational time remains fixed for variable Sppp and is mainly related to the size of the image. Finally, Fig. 6 shows examples of the estimated depth images indicating sharp and accurate results for the proposed strategy. This figure shows that UA over-smooths images while MANIPOP, which is designed for multi-peak scenarios, is more conservative leading to a number of "empty" pixels where no depth value can be estimated, especially in the extremely noisy cases. Furthermore, the proposed algorithm provides uncertainty estimates regarding the depth estimates as indicated by ϵ maps in the bottom row. It is shown that highest variances (i.e., uncertainty) are located near the edges or in presence of strong outliers. For completeness of this study, the figure also shows the results of the recent RT3D algorithm designed to deal with multi-peak scenarios [20], which is also conservative and results in a number of empty pixels where a depth value cannot be estimated. Note, however, that RT3D differs from the other algorithms used in this comparison as it processes a point cloud instead of an image, which makes a direct comparison more difficult. Note also that the obtained point cloud has been projected to the plan X-Y to obtain the images in Fig. 6, where white regions mean absence of detection. Fig. 7 shows the estimated point clouds at Sppp$= 0.4$, indicating that UA over-smooths the surface and connects edges, NR3D shows noisy results, while MNR3D shows the best compromise for this extreme case in addition to uncertainty maps to help select reliable estimates.

As previously indicated, we compared M2R3D with the Lindell denoising algorithm [32] on $185 \times 232 \times 1024$ histogram cubes with uniform background (generated using the [32] provided simulator). Fig. 8 shows the obtained true positive percentage w.r.t. distances for different Sppp, Bppp levels using M2R3D and Lindell algorithm. This figure shows that M2R3D reaches higher percentages faster, indicating a good generalizability of the proposed algorithm to other data format. An example of the estimated depth maps at the extreme case of Sppp=2 and Bppp=5 is shown in Fig. 9. It is clear that M2R3D

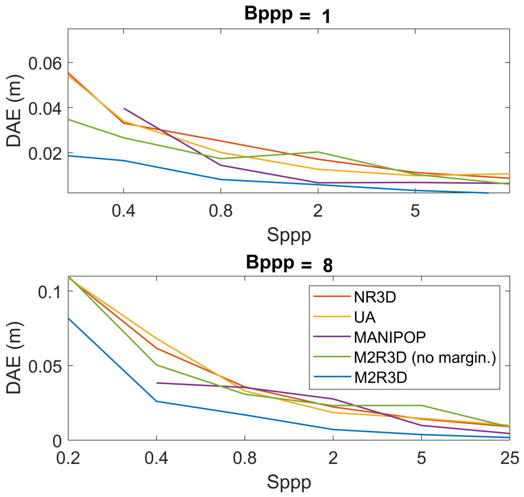

Fig. 5. Depth DAE (in metres) versus the signal average returns (Sppp) obtained with different algorithms for the bowling data generated with a uniform background level. MANIPOP's DAE is only computed for non-empty pixels.

TABLE I
COMPUTATIONAL TIME (IN SECONDS) OF THE COMPARED ALGORITHMS ON $123 \times 139 \times 300$ DATA CUBE GENERATED WITH A UNIFORM BACKGROUND.

		Sppp					
		0.2	0.4	0.8	2	5	25
Bppp=1	UA	9.0	9.1	9.2	11.3	13.8	15.3
	NR3D	190	200	156	69.1	45.6	33.4
	MANIPOP	43.2	137	133	167	144	162
	M2R3D	**3.7**	**3.6**	**3.6**	**3.6**	**3.6**	**3.6**
Bppp=8	UA	62.5	62.8	57.5	51.0	56.4	58.1
	NR3D	225	212	172	64.3	45.5	20.9
	MANIPOP	51.3	126	134	162	157	165
	M2R3D	**3.6**	**3.6**	**3.6**	**3.6**	**3.6**	**3.6**

preserves better object edges, can estimate small objects (e.g., see paint brushes) and is less sensitive to the intensity values (e.g., see bottom left region in Lindell map).

The second scenario is more challenging as it shows non-uniform background with respect to time, and has more photon detection which penalizes the UA and MANIPOP whose complexity is proportional to the number of detected photons. MANIPOP was not considered in this scenario due to its high computational cost and that it was specifically designed only to deal with the sparse photon regime. Fine tuning RT3D hyperparameters was not successful for this data and thus it is not considered. Fig. 10 shows the depth DAE obtained with the different algorithms, where the best results are obtained with the proposed strategy. Table II also indicates that the proposed algorithm is the fastest in this photon-dense regime. Finally, Fig. 11 shows examples of the estimated depth images, where UA and NR3D fail in the presence of high non-uniform background that leads to a large fraction of pixels failing to register a depth measurement, especially at low values of Sppp. Again the proposed algorithm highlights higher uncertainty around the edges, while showing low standard-deviation for other regions indicating good confidence in the provided estimates.

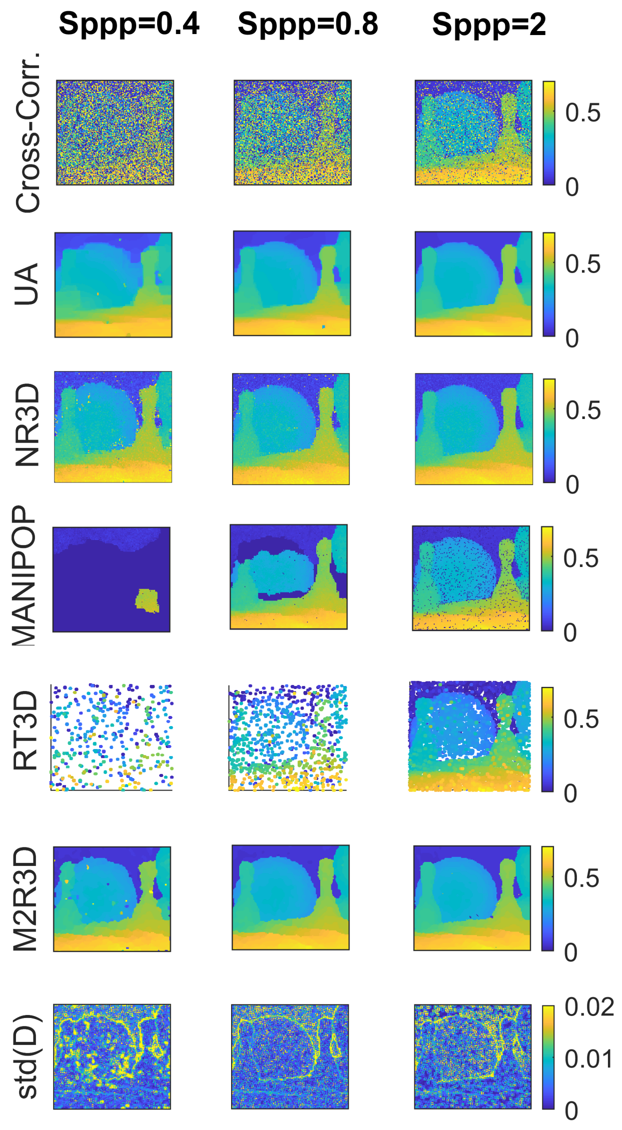

Fig. 6. Example of depth images estimated with different algorithms for the bowling data generated with a uniform background with an average of 1 photon count per-pixel (i.e., Bppp= 1). Uncertainty estimates (depth standard deviations) for the proposed M2R3D algorithm are shown in the bottom row.

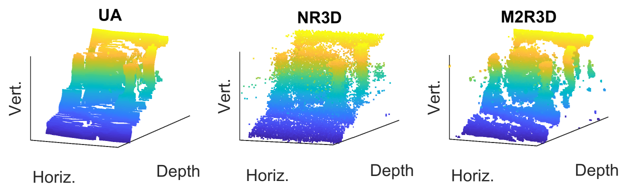

Fig. 7. Example of point clouds estimated with different algorithms for the bowling data generated with a uniform background, at Sppp= 0.4 and Bppp= 1.

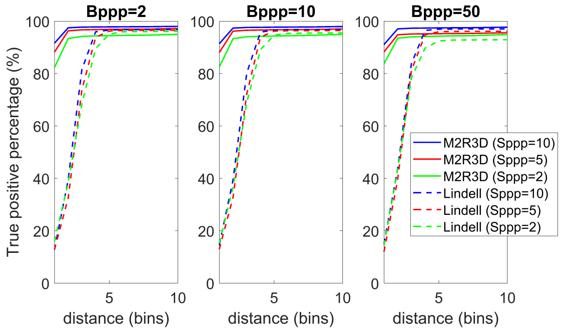

Fig. 8. True positive percentage w.r.t. distance (in bins) for different Bppp and Sppp levels. (Continuous lines) M2R3D, (dashed lines) Lindell algorithm.

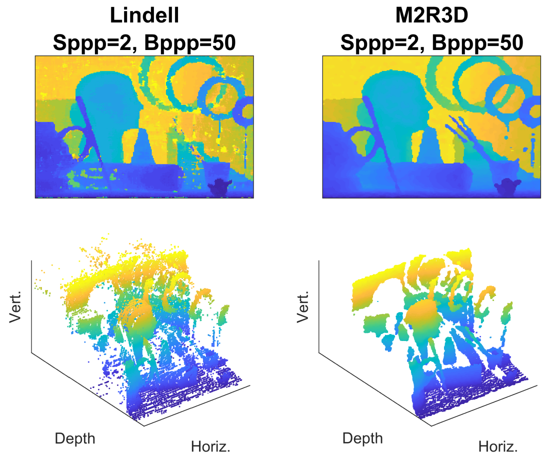

Fig. 9. Estimated depth maps for Lindell and M2R3D algorithms at Bppp=50 and Sppp=2. (Left column) Lindell algorithm, (right) M2R3D algorithm.

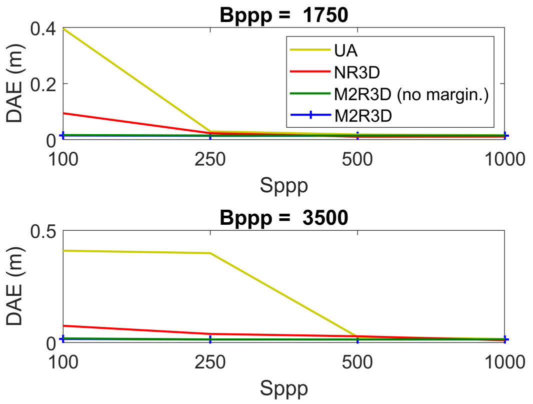

Fig. 10. Depth DAE (in meters) obtained with different algorithms for the bowling data generated with an exponentially decaying background level. (Top) average of 1750 background counts per-pixel, (bottom) average of 3500 background counts per-pixel.

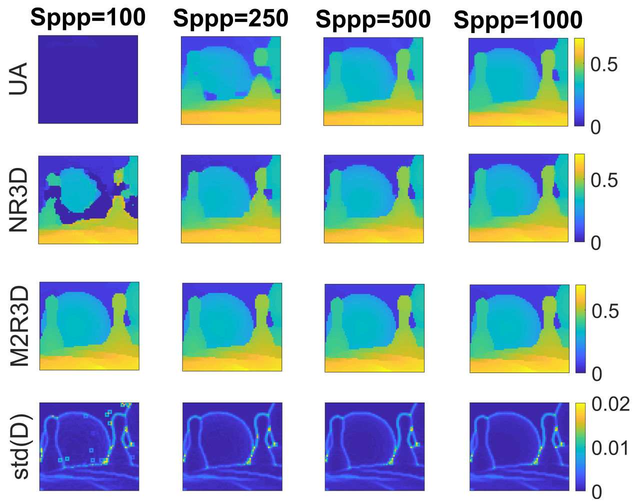

Fig. 11. Example of depth images estimated with different algorithms for the bowling data generated with an exponential background, at an average of 1750 background counts per-pixel. Uncertainty estimates (depth standard deviations) for the proposed M2R3D algorithm are shown in the bottom row.

TABLE II
COMPUTATIONAL TIME (IN SECONDS) OF THE COMPARED ALGORITHMS ON 62 × 70 × 340 DATA CUBE GENERATED WITH AN EXPONENTIAL BACKGROUND.

		Sppp			
		100	250	500	1000
Bppp=1750	UA	268.0	264.7	275.8	277.1
	NR3D	52.7	33.4	4.9	4.9
	M2R3D	**0.8**	**0.7**	**0.7**	**0.7**
Bppp=3500	UA	732.0	742.2	740.9	729.0
	NR3D	33.4	33.6	31	4.9
	M2R3D	**0.8**	**0.6**	**0.6**	**0.6**

V. RESULTS ON REAL DATA

This section evaluates quantitatively the results of the proposed algorithm on real data. We first provide more details regarding the real data considered in the main paper. Table III indicates the average signal and background levels obtained by M2R3D for Figs. 5, 6, and 7 of the main paper for the pixels located on the planar board targets and satisfying a depth error lower than 5.6cm (+- 1.5 time bin). The averaged SBR=Sppp/Bppp is also indicated together with the minimum and maximum values, for completeness. These SBR values are averaged across all pixels on the four wooden panels, with considerable variability being observed between individual pixels, as outlined in Table III . Note that these results indicate a high uncertainty on attenuation length (AL) especially for the measurements at high obscurant densities at 150m which show very low Sppp levels. These results indicate a AL level which may be even higher than stated in the main paper, AL was estimated using a different method. In order to retain statistical accuracy and avoid the deleterious effects of pulse pile-up [34]–[36], the time-correlated single-photon technique requires the photon count rate to be much lower than the excitation pulse rate. In the case of the measurements presented in this paper, none of the data sets were in the pulse pile-up regime. For example, in the results shown in Figure 5 in the main paper, the worst-case data set had an average per-pixel count rate that was 4% of the pulse rate. The maximum per-pixel value in this worst-case data set was 7%.

TABLE III
INDICATIVE AVERAGE SIGNAL AND BACKGROUND LEVELS ON REAL DATA.

4 boards target at 50m (Fig. 5 in the main paper), on 1024 pixels	Sppp	25	42	111	264
	Bppp	4037	5257	5854	5651
	SBR	0.006	0.008	0.019	0.046
	Min SBR	0.003	0.003	0.005	0.007
	Max SBR	0.010	0.019	0.038	0.105
	% Valid pixels	17	74	95	98
Target at 150m (Fig. 6 in the main paper), on 255 pixels	Sppp	5	86	1304	8006
	Bppp	3303	3766	5651	9092
	SBR	0.0015	0.023	0.231	0.880
	Min SBR	0.0008	0.0012	0.0045	0.0116
	Max SBR	0.004	0.071	0.521	2.202
	% Valid pixels	30	92	95	100
Moving target at 50m (Fig. 7 in the main paper), on 273 pixels	Sppp	459	656	420	
	Bppp	1365	1377	1342	
	SBR	0.337	0.476	0.313	
	Min SBR	0.044	0.082	0.043	
	Max SBR	0.820	1.130	0.767	
	% Valid pixels	100	100	100	

Second, we consider imaging the 3D wooden panel target (described in the main paper) located at a distance of 50m from the LiDAR system. The criterion used is the true positive percentage representing the percentage of pixels satisfying a given depth absolute error (DAE), where the reference depth is obtained by considering the low resolution depth image of the proposed algorithm in clear conditions (i.e., very low level of obscurant). Fig. 12 (top) represents the true positive percentage with respect to (top) time in seconds for a fixed DAE = 5.6cm and (bottom) depth absolute error in cm for the frame time=208s. The dashed lines in Fig. 12 (top) represents the time instants of the four images shown in Fig. 4 of the main document, i.e., 201s, 208s, 217s, 323s. This figure shows higher percentages obtained for the proposed M2R3D which quantitatively highlights the benefit of the proposed strategy on real data. It is shown in Fig. 12 (top) that M2R3D reached a true positive percentage > 90% level after 210s, advancing the other algorithms by ≈100s. Fig. 12 (bottom) also shows that M2R3D presents a true positive percentage>70% which is only reached by other algorithms when allowing a 80cm absolute depth error (i.e., DAE=0.8m).

Supplementary material 2: System description

A diagram of the bi-static LiDAR imaging system is shown in Supplementary Fig. 13. The system was mounted on a breadboard set-up having pitch and yaw adjustments, thus facilitating the alignment of the LiDAR system to the scene of interest.

On the illumination channel, the output fibre from the laser module was connected to a reflective collimation package and the collimated beam was passed through a 9 nm wide full-width half-maximum (FWHM) bandpass filter (BP2) with a central wavelength matched to that of the pulsed laser source. This was used to remove any potential out-of-band light or

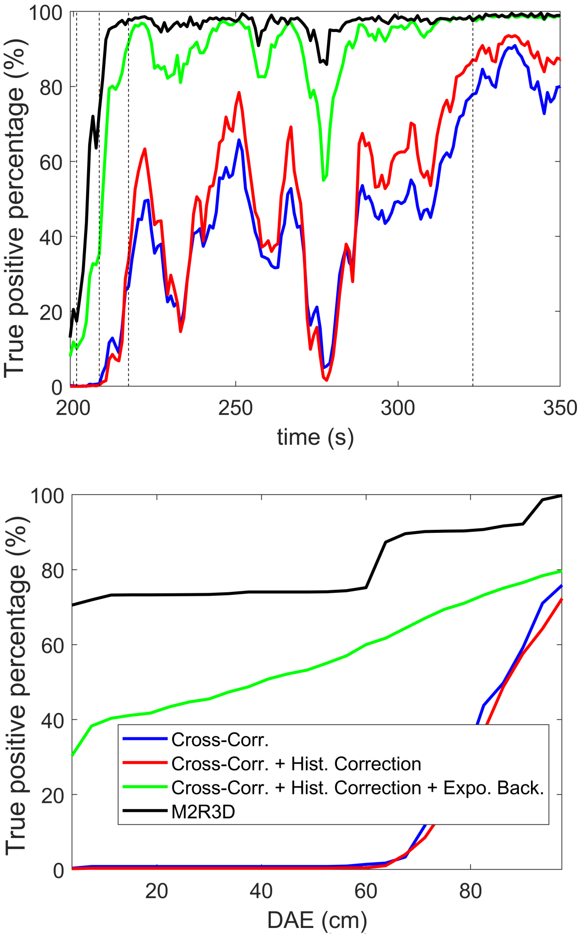

Fig. 12. True positive percentage w.r.t. (top) time in seconds for an DAE = 5.6cm (+- 1.5 time bin) and (bottom) depth absolute error in cm for time = 208s. The vertical dashed lines represent the time instants of the images of Fig. 4 in the main document. The true positive percentage is shown for the following algorithms: (a) Cross-correlation (blue line); (b) cross-correlation and histogram correction (red); (3) cross-correlation, histogram correction and exponential background (green); (4) M2R3D algorithm (black).

amplified spontaneous emission from the laser source. A pair of lenses in an adjustable telescope arrangement was used to control the divergence of the outgoing laser beam and thus the extent of the flood illumination at the target scene. These achromatic lenses were optimised for use in the SWIR region of the spectrum.

Controlled adjustments to the pitch and yaw of the optical arrangement for the illumination channel enabled the beam to be accurately positioned with respect to the field-of-view (FoV) of the 32 x 32 SPAD detector array. A combination of a longpass filter with a cut-on wavelength of 1500 nm (LP1), and a 9 nm FWHM bandpass filter similar to that used in the illumination channel, was inserted between OBJ1 and the SPAD detector array to limit out-of-band ambient light detection.

Each SPAD detector in the array had a quoted single photon detection efficiency of approximately 24% and a measured dark count rate averaging approximately 320 kcps (kilo-counts per second). A VIS-SWIR digital camera (Ninox 640, Raptor

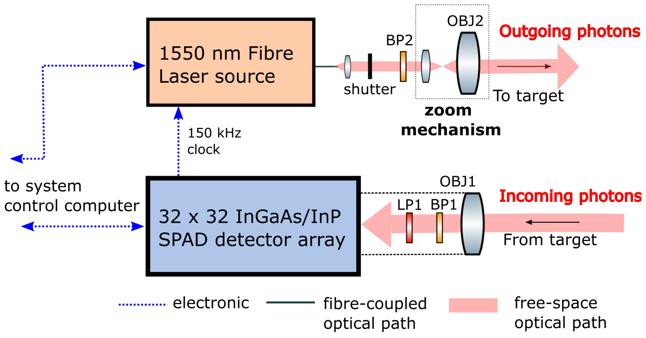

Fig. 13. A schematic diagram of the bi-static LiDAR imaging system configuration which comprised a λ = 1550 nm pulsed fibre laser source and a 32 x 32 InGaAs/InP SPAD detector array. Optical components include: objective lenses for both the detection (OBJ1) and transmission (OBJ2) channels; a longpass filter (LP1); and bandpass filters (BP1, BP2). Details on the high-performance optical filters and the zoom mechanism are given in the text.

Photonics) based on a 640×512 array of InGaAs PIN photodiode detectors was also mounted on the system breadboard and used for checking the alignment of the illumination and SPAD detector array camera channels to the scene of interest, and for monitoring the scene during a measurement. A 1500 nm long-pass spectral filter was used to limit the spectral range detected by this VIS-SWIR camera.

References

[1] J. Rapp and V. K. Goyal, "A few photons among many: Unmixing signal and noise for photon-efficient active imaging," *IEEE Trans. Comput. Imaging*, vol. 3, no. 3, pp. 445–459, Sept. 2017.

[2] Y. Altmann, X. Ren, A. McCarthy, G. S. Buller, and S. McLaughlin, "Lidar waveform based analysis of depth images constructed using sparse single photon data," *IEEE Trans. Image Process.*, vol. 25, no. 5, pp. 1935–1946, Mar. 2015.

[3] Y. Altmann and S. McLaughlin, "Range estimation from single-photon lidar data using a stochastic em approach," in *EUSIPCO-18*, Rome, Italy, Sept. 2018, pp. 1112–1116.

[4] M. Denitto, M. Bicego, A. Farinelli, and M. Figueiredo, "Spike and slab biclustering," *Pattern Recognition*, vol. 72, pp. 186 – 195, 2017.

[5] M. Pfennigbauer, C. Wolf, J. Weinkopf, and A. Ullrich, "Online waveform processing for demanding target situations," in *Laser Radar Technology and Applications XIX; and Atmospheric Propagation XI*, M. D. Turner, G. W. Kamerman, L. M. W. Thomas, and E. J. Spillar, Eds., vol. 9080, International Society for Optics and Photonics. SPIE, 2014, pp. 142 – 151.

[6] G. Satat, M. Tancik, and R. Raskar, "Towards photography through realistic fog," in *Computational Photography (ICCP), 2018 IEEE International Conference on*. IEEE, 2018, pp. 1–10.

[7] M. Henriksson, L. Allard, and P. Jonsson, "Panoramic singleâĂŠphoton counting 3D lidar," in *Electro-Optical Remote Sensing XII*, G. Kamerman and O. Steinvall, Eds., vol. 10796, International Society for Optics and Photonics. SPIE, 2018, pp. 33 – 45.

[8] A. Halimi, Y. Altmann, A. McCarthy, X. Ren, R. Tobin, G. S. Buller, and S. McLaughlin, "Restoration of intensity and depth images constructed using sparse single-photon data," in *Proc. EUSIPCO*, 2016, pp. 86–90.

[9] Y. Altmann, S. McLaughlin, and M. E. Davies, "Fast online 3d reconstruction of dynamic scenes from individual single-photon detection events," *IEEE Transactions on Image Processing*, vol. 29, pp. 2666–2675, 2020.

[10] J. Tachella, Y. Altmann, X. Ren, A. McCarthy, G. S. Buller, S. McLaughlin, and J.-Y. Tourneret, "Bayesian 3d reconstruction of complex scenes from single-photon lidar data," *SIAM Journal on Imaging Sciences*, vol. 12, no. 1, pp. 521–550, 2019.

[11] W. Marais and R. Willett, "Proximal-gradient methods for poisson image reconstruction with bm3d-based regularization," in *2017 IEEE 7th International Workshop on Computational Advances in Multi-Sensor Adaptive Processing (CAMSAP)*, 2017, pp. 183–187.

[12] A. Halimi, R. Tobin, A. McCarthy, J. Bioucas-Dias, S. McLaughlin, and G. S. Buller, "Robust restoration of sparse multidimensional single-photon lidar images," *IEEE Trans. Comput. Imaging*, vol. 6, pp. 138–152, 2020.

[13] O. Dikmen and A. Cemgil, "Gamma markov random fields for audio source modeling," *IEEE Trans. Audio, Speech, Language Process.*, vol. 18, no. 3, pp. 589–601, March 2010.

[14] T. Park and G. Casella, "The bayesian lasso," *Journal of the American Statistical Association*, vol. 103, no. 482, pp. 681–686, 2008.

[15] M. A. T. Figueiredo, "Adaptive sparseness for supervised learning," *IEEE Transactions on Pattern Analysis and Machine Intelligence*, vol. 25, no. 9, pp. 1150–1159, 2003.

[16] D. P. Bertsekas, *Nonlinear programming*. Belmont, Massachusetts: Athena Scientific, 1995.

[17] J. Sigurdsson, M. Ulfarsson, and J. Sveinsson, "Hyperspectral unmixing with l_q regularization," *IEEE Trans. Geosci. Remote Sens.*, vol. 52, no. 11, pp. 6793–6806, Nov. 2014.

[18] Y. Romano, M. Elad, and P. Milanfar, "The little engine that could: Regularization by denoising (red)," *SIAM Journal on Imaging Sciences*, vol. 10, no. 4, pp. 1804–1844, 2017.

[19] S. V. Venkatakrishnan, C. A. Bouman, and B. Wohlberg, "Plug-and-play priors for model based reconstruction," in *2013 IEEE Global Conference on Signal and Information Processing*, 2013, pp. 945–948.

[20] J. Tachella, Y. Altmann, N. Mellado, A. McCarthy, R. Tobin, G. S. Buller, J.-Y. Tourneret, and S. J. McLaughlin, "Real-time 3d reconstruction from single-photon lidar data using plug-and-play point cloud denoisers," *Nature Communications*, vol. 10, no. 1, p. 4984, 2019.

[21] S. Boyd, N. Parikh, E. Chu, B. Peleato, and J. Eckstein, "Distributed optimization and statistical learning via the alternating direction method of multipliers," *Found. Trends Mach. Learn.*, vol. 3, no. 1, pp. 1–122, Jan 2011.

[22] Q. Zhang, L. Xu, and J. Jia, "100+ times faster weighted median filter (wmf)," in *2014 IEEE Conference on Computer Vision and Pattern Recognition*, June 2014, pp. 2830–2837.

[23] W. Liu, X. Chen, J. Yang, and Q. Wu, "Robust color guided depth map restoration," *IEEE Trans. Image Process.*, vol. 26, no. 1, pp. 315–327, 2017.

[24] S. Chan, A. Halimi, F. Zhu, I. Gyongy, R. K. Henderson, R. Bowman, S. McLaughlin, G. S. Buller, and J. Leach, "Long-range depth imaging using a single-photon detector array and non-local data fusion," *Scientific Reports*, vol. 9, pp. 8075, number = 1, available = y, topic = , 2019.

[25] X. Liu, D. Zhai, R. Chen, X. Ji, D. Zhao, and W. Gao, "Depth super-resolution via joint color-guided internal and external regularizations," *IEEE Transactions on Image Processing*, vol. 28, no. 4, pp. 1636–1645, April 2019.

[26] D. Shin, A. Kirmani, V. K. Goyal, and J. H. Shapiro, "Photon-efficient computational 3-d and reflectivity imaging with single-photon detectors," *IEEE Transactions on Computational Imaging*, vol. 1, no. 2, pp. 112–125, 2015.

[27] P. Milanfar, "A tour of modern image filtering: New insights and methods, both practical and theoretical," *IEEE Signal Processing Magazine*, vol. 30, no. 1, pp. 106–128, 2013.

[28] K. He, J. Sun, and X. Tang, "Guided image filtering," *IEEE Transactions on Pattern Analysis and Machine Intelligence*, vol. 35, no. 6, pp. 1397–1409, June 2013.

[29] D. Ferstl, C. Reinbacher, R. Ranftl, M. Ruether, and H. Bischof, "Image guided depth upsampling using anisotropic total generalized variation," in *2013 IEEE International Conference on Computer Vision*, Dec 2013, pp. 993–1000.

[30] C. Guo, C. Li, J. Guo, R. Cong, H. Fu, and P. Han, "Hierarchical features driven residual learning for depth map super-resolution," *IEEE Transactions on Image Processing*, vol. 28, no. 5, pp. 2545–2557, May 2019.

[31] K. Zhou, S. Yu, and C. Jung, "Alternately guided depth super-resolution using weighted least squares and zero-order reverse filtering," in *ICASSP 2019 - 2019 IEEE International Conference on Acoustics, Speech and Signal Processing (ICASSP)*, May 2019, pp. 1847–1851.

[32] D. B. Lindell, M. O'Toole, and G. Wetzstein, "Single-photon 3d imaging with deep sensor fusion," *ACM Trans. Graph.*, vol. 37, no. 4, pp. 113:1–113:12, July 2018.

[33] D. Scharstein and C. Pal, "Learning conditional random fields for stereo," in *2007 IEEE Conference on Computer Vision and Pattern Recognition*, June 2007, pp. 1–8.

[34] W. Becker, *Advanced Time-Correlated Single Photon Counting Techniques*. Springer-Verlag Berlin Heidelberg, 2005.

[35] A. Gupta, A. Ingle, A. Velten, and M. Gupta, "Photon-flooded single-photon 3d cameras," in *ArXiv e-prints*, Apr. 2019.

[36] A. K. Pediredla, A. C. Sankaranarayanan, M. Buttafava, A. Tosi, and A. Veeraraghavan, "Signal processing based pile-up compensation for gated single-photon avalanche diodes," in *ArXiv e-prints*, Jun. 2018.
